# Podocyte-directed *VEGFC* gene therapy prevents increased glomerular permeability and glycocalyx damage in experimental type 1 diabetes

**DOI:** 10.1016/j.ymthe.2025.10.001

**Published:** 2025-10-06

**Authors:** Aldara Martin Alonso, Carl J. May, Holly Stowell-Connolly, Haijie Wu, Monica Gamez, Khadija Ourradi, Raina D. Ramnath, Wen Yi Ding, Gavin I. Welsh, Simon C. Satchell, Rebecca R. Foster

**Affiliations:** 1 Bristol Renal, Translational Health Sciences, Bristol Medical School, University of Bristol, Bristol BS1 3NY, UK

**Keywords:** VEGFC, gene therapy, diabetic kidney disease, glycocalyx, albuminuria, glomerulus, type 1 diabetes

## Abstract

Diabetic kidney disease (DKD) is the leading cause of end-stage renal failure, and current interventions fail to directly target the glomerulus, where the disease initiates. Vascular endothelial growth factor (VEGF)C is a key contributor to glomerular endothelial barrier function. In transgenic mice, podocyte-specific overexpression of human VEGFC was protective in early DKD. Here, we investigated the therapeutic potential of a podocyte-targeted *VEGFC* gene therapy in DKD. We employed an adeno-associated virus (AAV2/9) to drive human VEGFC in human and mouse podocytes. Expressed VEGFC was functional *in vitro*. In type 1 diabetic mice (induced by streptozotocin), systemic administration of AAV2/9 increased glomerular human VEGFC expression, ameliorating both albuminuria and increased glomerular permeability. Importantly, *VEGFC* gene therapy also protected the glomerular endothelial glycocalyx, the first barrier to protein in the glomerular filtration barrier. These findings demonstrate that podocyte-directed *VEGFC* gene delivery can restore glomerular function and protect against early DKD progression. This novel approach represents a promising therapeutic strategy, particularly for patients with type 1 diabetes at risk of DKD, where there is an unmet clinical need.

## Graphical abstract

**Figure:**
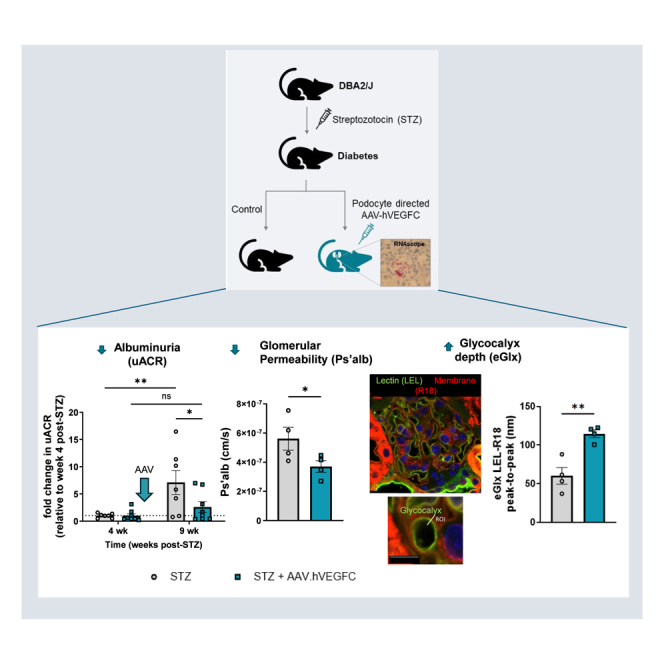

## Teaser

There is a critical need for novel interventions that can slow the progression of kidney disease in diabetes, particularly in type 1 diabetes. Here, Foster and colleagues show that gene therapy is a viable option for targeted delivery in the kidney in diabetes, using adeno-associated virus to deliver a growth factor (VEGFC).

## Introduction

Diabetic kidney disease (DKD) is the most prevalent long-term complication of diabetes and the leading cause of end-stage renal disease in the Western world. The incidence of DKD is expected to rise, as global diabetes cases have quadrupled globally since 1990 and could more than double by 2050.^1^ In type 1 diabetes (T1D), up to 30% of these individuals develop DKD within 10 years of diagnosis.^2^ The UK was ranked fifth globally for T1D incidence among children aged 0–14 years, with a rate of 25 cases per 100,000 children, in 2021 (NHS England). Once DKD progresses, kidney damage is irreversible, limiting treatment options to dialysis or a kidney transplant. These treatments not only diminish patients’ quality of life, but also impose a substantial economic burden. DKD accounts for 21% of deaths in people with T1D and 11% in those with type 2 diabetes (T2D).^3^ Recent renoprotective therapeutic advances for T2D have not been approved for T1D, highlighting a significant unmet clinical need in patients with T1D at risk of DKD.

Increasing albuminuria is the earliest indicator of DKD, reflecting damage to the glomerular endothelium before ultrastructural changes in podocytes are observed.^4^ Diabetes induces glomerular endothelium dysfunction, particularly by damaging the proteoglycan-rich layer coating the glomerular endothelium, known as the endothelial glycocalyx (eGlx). Despite this, glomerular endothelial-specific therapies for DKD have not been developed. Glomerular vascular endothelial growth factor (VEGF)C, expressed by podocytes, enhances the glycocalyx barrier properties of the glomerular endothelium.^5^^,^^6^^,^^7^ Using a transgenic mouse model, we have demonstrated the therapeutic potential of podocyte-specific human VEGFC overexpression in experimental DKD.^7^ Herein, we take the next step toward translation, hypothesizing that *VEGFC* gene therapy will ameliorate albuminuria in a T1D model. This advanced approach employs an adeno-associated virus (AAV)-based gene therapy tool that can effectively target podocytes.^8^

## Results

### AAV induces the expression of functional human VEGFC in podocytes *in vitro*/*ex vivo* using a minimal nephrin promoter

**Figure 1:**
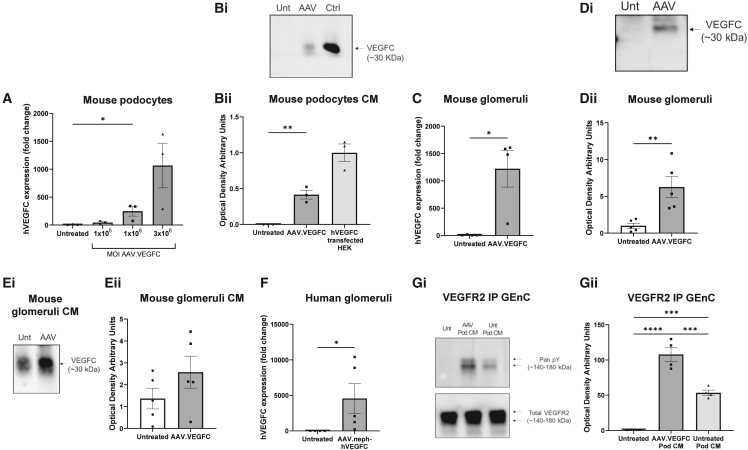
AAV induces the expression of functional human VEGFC in podocytes *in vitro*/*ex vivo* using a minimal nephrin promoter Conditionally immortalized mouse podocytes (Pods) were infected with AAV.neph-*VEGFC* (AAV.*VEGFC*) (manufactured in-house) at different multiplicity of infection (MOI) doses, and transduction of human *VEGFC* mRNA was shown by qPCR (A). Expression is relative to mouse GAPDH and untreated (Unt) control (2^−ΔΔCt^). Unt, *n* = 3; AAV.*VEGFC*, *n* = 3 (Kruskal-Wallis test, *p* < 0.01, followed by Dunn’s multiple comparison test of Unt vs. MOI 1 × 10^6^). (B) Detection of human VEGFC protein in concentrated conditioned medium (CM) from mouse Pods transduced with AAV.*VEGFC* (MOI 1 × 10^6^; manufactured in-house) by western blotting. HEK293T cells were transfected with a plasmid driving *VEGFC* expression under the cytomegalovirus (CMV) promoter as a positive control of the blot. A representative blot is shown (Bi), and data are summarized (Bii). Unt, *n* = 3; AAV.*VEGFC* (AAV), *n* = 3; hVEGFC-transfected HEK (Ctrl), *n* = 3 (one-way ANOVA, *p* < 0.001, followed by Šídák’s multiple comparisons test of Unt vs. AAV-transduced podocyte CM). Isolated murine glomeruli from wild-type SV129 mice were transduced with AAV.neph-*VEGFC* (MOI 1 × 10^5^; manufactured by VectorBuilder), and expression of *VEGFC* was measured on day 5 after *ex vivo* infection by qPCR (C; Unt, *n* = 4; AAV.neph-*VEGFC*, *n* = 4; one-tailed Mann-Whitney test), by western blotting of the glomeruli; a representative blot is shown (Di), and data are summarized (Dii) (Unt, *n* = 5; AAV.neph-*VEGFC*, *n* = 5 [unpaired *t* test]), and of the CM; a representative blot is shown (Ei), and data are summarized (Eii) (Unt, *n* = 5; AAV.neph-*VEGFC*, *n* = 5 [unpaired *t* test]). (F) hVEGFC qPCR on day 5 post-*ex*-*vivo* transduction of isolated human glomeruli with AAV.*VEGFC* (MOI 1 × 10^5^; ultrapure particles manufactured by VectorBuilder). Unt, *n* = 4; AAV.*VEGFC*, *n* = 5 (unpaired *t* test). (Gi) Immunoprecipitation (IP) with anti-VEGFR2 antibody of conditionally immortalized human glomerular endothelial cells (GEnCs) treated for 15 min with CM from mouse Pods previously transduced with AAV.*VEGFC* (manufactured in-house) or Unt (10 days) and western blotting with a pan anti-phosphotyrosine antibody (pan pY) or anti-VEGFR2. Unt GEnC (Unt), *n* = 4; AAV.*VEGFC* Pod CM (AAV Pod CM), *n* = 4; Unt Pod CM (Unt Pod CM), *n* = 4. (Gii) Optical density normalized to total VEGFR2 and relative to Unt GEnC (one-way ANOVA followed by Tukey’s multiple comparisons test, *p* < 0.0001). All data are presented as mean ± SEM. ∗*p* < 0.05, ∗∗*p* < 0.01, ∗∗∗*p* < 0.001, and ∗∗∗∗*p* < 0.0001.

AAV.neph-*VEGFC* was initially manufactured in-house. Mouse conditionally immortalized podocytes were transduced with AAV.neph-*VEGFC* particles to confirm *VEGFC* expression. *VEGFC* mRNA expression, quantified by RT-qPCR, significantly increased in a dose-dependent manner in podocytes transduced at different multiplicities of infection (MOIs) of the AAV (Figure 1A). hVEGFC was measured in the conditioned media of AAV-transduced mouse podocytes by western blotting, using media from HEK (human embryonic kidney) cells previously transfected with a *VEGFC*-expressing plasmid as positive controls (Figure 1Bi). Secreted hVEGFC expression was significantly higher in AAV-transduced podocytes compared to untreated podocytes (Figure 1Bii). Isolated wild-type mouse glomeruli were transduced with AAV.neph-*VEGFC* particles. At day 5 post-AAV transduction, mouse glomeruli showed a significant increase in *VEGFC* mRNA (Figure 1C) and protein expression by western blotting (Figures 1Di and 1Dii) and a trend toward increased VEGFC secretion, as shown by western blotting of the conditioned media (Figures 1Ei and 1Eii) that peaked at day 4 (Figure S1). Importantly, AAV-transduced isolated human glomeruli also showed a significant increase in *VEGFC* mRNA expression (Figure 1F). Thus, we have demonstrated successful gene delivery to *ex vivo* human glomeruli and demonstrated that the AAV2/9 serotype was effective for both mouse and human tissue.

To demonstrate the functionality of AAV-induced hVEGFC, human conditionally immortalized glomerular endothelial cells (GEnCs) were treated with conditioned media from AAV-transduced mouse podocytes, and activation of the endothelial-specific receptor tyrosine kinase VEGFR receptor 2 (VEGFR2) was assessed. This activation is crucial for mediating VEGFC’s effects on GEnC barrier properties.^5^^,^^7^ GEnC protein was immunoprecipitated using an anti-VEGFR2 antibody, and then phosphorylated tyrosine (pY) and total VEGFR2 were detected by western blotting (Figure 1Gi). Conditioned media from AAV-treated podocytes significantly increased pY in GEnCs at the same molecular weight as VEGFR2, indicating that secreted hVEGFC triggers VEGFR2 phosphorylation in GEnC (Figure 1Gii).

### AAV.neph-VEGFC ameliorates albuminuria and restores the glomerular eGlx in T1D

**Figure 2:**
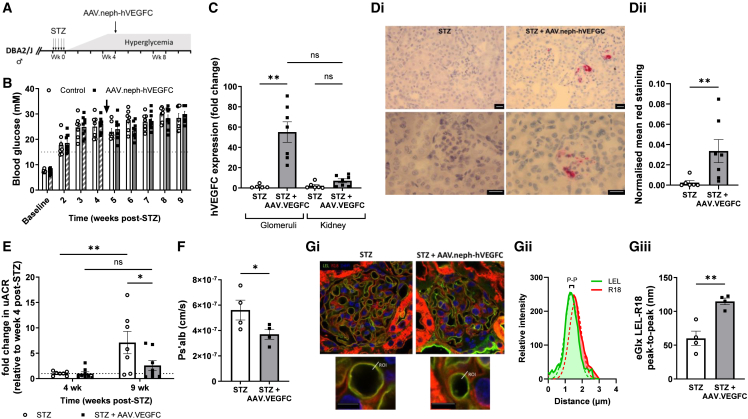
AAV.neph-*VEGFC* ameliorates albuminuria and restores the glomerular endothelial glycocalyx in type 1 diabetes DBA2/J male mice were given streptozotocin (STZ) during week 0 and then AAV.neph-*VEGFC* (AAV.*VEGFC*) (ultrapure particles manufactured by VectorBuilder) by tail vein injection (dose: 7.50 × 10^13^ GC/kg of body weight) at week 4 (A). Mice were terminated at week 9–10 post-STZ administration. (B) Weekly blood glucose (if out of range [>33.3 mM], it was plotted as 33.3 mM). Grid line: 15 mM threshold. Arrow: virus injection 4 weeks following STZ induction. STZ, *n* = 7; STZ + AAV.*VEGFC*, *n* = 9 (two-way ANOVA, *p <* 0.0001). (C) *VEGFC* qPCR of isolated glomeruli and kidney (week 9–10 post-STZ). Expression is relative to mouse GAPDH and control mice (2^−ΔΔCt^). STZ, *n* = 5–6; STZ + AAV.*VEGFC*, *n* = 7. Kruskal-Wallis test (*p <* 0.001) followed by Dunn’s multiple comparison tests. (Di) Kidney sections were subjected to chromogenic (red) *in situ* hybridization of WPRE (under the control of a nephrin promoter, such as *VEGFC*). Magnifications: 20× (top) and 40× (bottom). Scale bar: 20 μm. (Dii) Glomerular red signal normalized to background. STZ, *n* = 6 animals; STZ + AAV.*VEGFC*, *n* = 7 animals (Mann-Whitney one-tailed test). (E) Urine albumin-to-creatinine ratio (uACR) relative to uACR at week 4 post-STZ (just before virus injection). STZ, *n* = 7; STZ + AAV*.VEGFC*, *n* = 8 (two-way ANOVA followed by Bonferroni’s multiple comparison test to compare animal groups or Šídák’s multiple comparisons test to compare time points within each animal group). Absolute uACR in Figure S2. (F) In cardiac perfused mice, sieved glomeruli were loaded with octadecyl rhodamine B chloride (R18) and Alexa Fluor 488-BSA and subjected to perfusion with unlabeled BSA to measure albumin permeability (Ps’alb). One-tailed unpaired *t* test (*p <* 0.05) of the average Ps’alb of animals (STZ [*n* = 4], *n* = 21 glomeruli; STZ + AAV.*VEGFC* [*n* = 4], *n* = 26 glomeruli). We have previously reported that DBA2/J male mice receiving vehicle (citrate buffer) instead of STZ have a Ps’alb of 3.144 ± 0.295 (cm/s × 10^−7^).^6^ In cardiac perfused mice, kidney sections were stained with FITC-labeled *Lycopersicon esculentum* lectin (LEL) and R18. Confocal representative images (magnification: 63×) (Gi). Top scale bar: 20 μm; bottom scale bar: 5 μm. ROI, region of interest used to measure the distance between the fluorophore profile peaks (peak-to-peak [P-P], Gii). P-P is an index of glycocalyx depth (one-tailed unpaired *t* test, Giii). Kidney sections from a separate batch of vehicle control DBA2/J male mice were subjected to the same processing and staining and show a P-P (132.0 ± 13.43; *n* = 5) significantly higher than the P-P of the STZ group (showing that STZ-induced diabetes reduces eGlx thickness) and non-significantly different from the P-P of STZ + AAV.VEGFC mice (one-way ANOVA, *p <* 0.01, followed by Bonferroni’s multiple comparison test, *p <* 0.01). All data are given as mean ± SEM. ∗*p <* 0.05, ∗∗*p <* 0.01, and ∗∗∗*p <* 0.001.

VEGFC gene therapy was applied to an experimental model of type 1 DKD (Figure 2A). Following streptozotocin (STZ) injection, all mice became hyperglycemic (Figure 2B). At 4 weeks post-STZ, mice received the AAV.neph-*VEGFC* via tail vein injection at 7.50 × 10^13^ genomic copies (GC)/kg, a dose based on Ding et al.^8^ The AAV intervention did not affect body weight (Figure S2). *VEGFC* mRNA expression significantly increased in sieved glomeruli, but not in the whole kidney (Figure 2C). Mirroring this, *in situ* hybridization of an AAV regulatory element, also driven by the nephrin promoter, demonstrated transgene expression specifically in the glomerulus (Figures 2Di and 2Dii). Transgene mRNA expression increased significantly in the liver (almost 2,000 times higher in AAV-treated mice) and to a lesser extent in the lung (Figure S3). Resident/infiltrating macrophages were shown to be unchanged between STZ and STZ+AAV.*VEGFC* mice in both glomerular and tubular regions (Figure S4). Of note, very few macrophages were quantified within glomeruli. *VEGFC* gene therapy was expected to prevent albuminuria and glomerular leakiness, as previously shown *ex vivo* and in transgenic mice.^7^ Importantly, AAV.neph-*VEGFC* significantly attenuated the diabetes-induced albuminuria (Figure 2E, fold change due to variability in urine albumin-to-creatinine ratio [uACR]; Figure S5, uACR) and glomerular albumin permeability (Figure 2F), a more sensitive and direct measure of glomerular filtration barrier integrity than uACR.^9^

We hypothesized that *VEGFC* gene therapy would prevent diabetes-induced GEnC barrier dysfunction, specifically through restoration of eGlx damage. EGlx components were labeled with FITC-labeled tomato lectin, and the endothelial membrane was labeled with octadecyl rhodamine B chloride (R18) (Figure 2Gi). A linear region of interest, from the luminal to the abluminal side of the glomerular capillary, provided fluorophore profiles, and the distance between the peaks of these profiles (lectin to R18, respectively) was used as a measure of eGlx depth (Figure 2Gii).^10^ EGlx depth was significantly increased in AAV-treated diabetic mice compared to untreated diabetic mice (Figure 2Giii).

## Discussion

We demonstrate that gene therapy can be used to target the glomerular filtration barrier in DKD, confirming the effectiveness of *VEGFC* gene therapy in T1D.

DKD is a progressive condition, and there are no available treatments that reverse existing kidney damage. Current pharmacological treatments, such as renin-angiotensin system inhibitors and sodium-glucose co-transporter 2 inhibitors, aim to manage underlying conditions to slow the renal function decline in T2D but have not been approved for patients with T1D. Gene therapies offer promising strategies to address this gap. Herein, we propose a podocyte-directed gene therapy using an AAV2/9 capsid in combination with a podocyte-specific promoter, a gene delivery approach effective in rescuing from podocin defects in models of nephrotic syndrome.^8^

It is well established that paracrine growth factors play crucial roles in the crosstalk between podocytes and the adjacent GEnCs to ensure adequate blood filtration at the glomerular filtration barrier. The VEGFC precursor undergoes a series of proteolytic processing steps that increase its affinity and ability to activate VEGFR3, and the fully processed mature form can also activate VEGFR2, as reported in endothelial cells *in vitro*.^11^ VEGFR2, which is expressed on the glomerular endothelium, is activated (phosphorylated) by recombinant VEGFC in cultured GEnCs.^6^ Here, we demonstrate that AAV-induced VEGFC is secreted from podocytes and activates VEGFR2 in cultured GEnC, suggesting functional hVEGFC. Previously, we showed that VEGFC increases monolayer integrity in cultured GEnC and counteracts the increased permeability of *ex vivo* glomeruli from T2D *db/db* mice.^5^^,^^7^ Inducing podocyte-specific VEFGC expression in transgenic mice reduced uACR in STZ-driven diabetes, both prophylactically and as an intervention.^7^ Importantly, we have now translated this into a *VEGFC* gene therapy that reduced uACR by ∼64%, as well as glomerular permeability, in T1D mice. Of note, a ≥30% reduction in albuminuria is recommended to slow chronic kidney disease progression by the American Diabetes Association.^12^

Podocyte-directed AAV-*VEGFC* also increased the depth of glycocalyx, which is damaged in humans with T1D, including in kidney biopsies, and in STZ-treated mice.^10^^,^^13^^,^^14^ This finding supports the growing evidence that targeting eGlx shedding is an effective strategy to mitigate the increased glomerular permeability and albuminuria in experimental DKD.^9^^,^^10^ VEGFC enhances the synthesis of sulfated glycosaminoglycans in cultured GEnC and restores impaired glomerular permeability and the eGlx damage caused by glycosaminoglycan-shedding enzymes.^6^^,^^7^ We have shown that AAV-induced VEGFC increased eGlx depth, thereby improving glomerular endothelial macromolecular barrier properties, leading to an overall reduction in albuminuria and glomerular permeability.

We suggest that chronic podocyte-specific VEGFC overexpression alters glomerular signaling of VEGFA_165_. VEGFA_165_, essential for maintaining the glomerular endothelium and normal blood filtration, is produced by podocytes and acts on GEnC mainly by activating VEGFR2.^15^ There are limitations in extrapolating these mouse models to human disease, as rodent models cannot fully recapitulate disease progression or severity. In particular, STZ-induced mice do not develop a reduction in the estimated glomerular filtration rate (eGFR) and therefore loss in renal function. However, increased levels of VEGFA and VEGFR2 are linked to human kidney disease, including early DKD.^16^^,^^17^ Using two human DKD datasets from the Gene Expression Omnibus database, upregulated VEGFA to VEGFR2 glomerular signaling was shown. Also, VEGFA expression correlated with increased eGFR (hyperfiltration, indicating early disease), but VEGFC did not.^18^ Importantly, these human datasets reflect changes that have been shown previously in diabetic rodent models. VEGFA_165_ and mature VEGFC compete for VEGFR2 binding in cultured endothelial cells.^11^ In cultured GEnC, recombinant VEGFA phosphorylates VEGFR2 more rapidly and to a higher degree than VEGFC and, unlike VEGFC, increases permeability.^5^ VEGFA also increased mouse glomeruli permeability *ex vivo*, which was ameliorated by VEGFC.^7^ In addition, and in contrast to VEGFC, VEGFA promoted shedding of charged glycosaminoglycans in cultured GEnC.^6^ At week 8, STZ diabetic mice showed increased glomerular VEGFA and VEGFR2 expression (VEGFC was unchanged), but this was attenuated in diabetic mice overexpressing VEGFC in podocytes.^7^ In addition to attenuating the diabetes-induced changes in VEGFA signaling, podocyte-specific VEGFC overexpression promoted VEGFR2/VEGFR3 heterodimerization in healthy transgenic mice.^7^ Thus, the AAV-induced VEGFC may attenuate VEGFA_165_ signaling by competing for VEGFR2 binding and activating different downstream signals through VEGFR3, resulting in reduced permeability and improved eGlx.

The use of VEGFC clinically has shown potential in breast cancer-related lymphedema using the adenovirus expressing *VEGFC*, Lymphactin. Importantly, we have shown that AAV.neph-*VEGFC* injection into wild-type mice is tolerated, and mice appear macroscopically normal. There are conflicts in the literature regarding VEGFC in disease. Circulating VEGFC has been significantly correlated with weight gain/metabolic syndrome,^19^ and overexpression of VEGFC in mouse skin led to elevated blood glucose and insulin resistance.^20^ However, previous work has also shown that circulating VEGFC is significantly inversely related to all-cause mortality, with low levels of serum VEGFC being an independent risk factor for cardiovascular death in patients with coronary artery disease, in a multicenter prospective cohort study (*n* = 2,418).^21^ In our STZ mice, we demonstrated that AAV.neph-*VEGFC* did not affect weight gain or induce hyperglycemia. VEGFC can act as a chemoattractant to macrophages under inflammatory conditions,^22^ but targeted delivery of VEGFC reduces immune cell density in diabetic wounds and helps to accelerate repair.^23^ We found no evidence of increased macrophage presence in the STZ mice treated with AAV.neph-*VEGFC*, suggesting that glomerular VEGFC does not attract macrophages.

While VEGFA inhibition is of interest in DKD as a potential therapy, it can have undesirable effects.^24^ Therapies that attenuate excessive VEGFA_165_ signaling without abolishing its essential functions are attractive. Systemic treatment with recombinant VEGFA_165_b, which binds to VEGFR2 with equal affinity to VEGFA_165_ but activates different downstream signaling in endothelial cells, restored eGlx and ameliorated DKD in mice.^14^ Blocking excessive VEGFR2 activation by systemic treatment with either a fragmented VEGFR2 antibody or a VEGFR2 kinase inhibitor has also shown promise in experimental DKD.^25^ In contrast to these strategies, our gene therapy approach aims to promote VEGFC signaling specifically in the glomerulus. While the half-life of recombinant VEGFC is short (<15 min) in mouse circulation,^26^ our gene therapy approach would overcome this, as AAVs achieve long-term transgene expression. This is primarily attributed to the stabilization of the AAV genome as circular double-stranded extrachromosomal DNA (episomes) that persist inside the nucleus of postmitotic cells, like podocytes. AAVs are also the preferred gene therapy vector for their minimal pathogenicity.

We believe *VEGFC* gene therapy may be particularly beneficial in patients with T1D at the early stage of DKD, when albuminuria is increasing. Although this study focused on DKD, cell-specific *VEGFC* gene therapy could be beneficial in other kidney diseases, such as polycystic kidney disease.^27^ We acknowledge that the transgene expression was not exclusive to the glomeruli, as anticipated with the dose and administration route.^8^ If a systemic route were to be pursued, then extensive safety profiling would be necessary in terms of liver function and systemic VEGFC expression. However, to avoid potential side effects of off-target expression, the next steps will involve using a direct delivery route with a lower virus titer. Targeted delivery in Gottingen minipigs, using AAV with a podocyte-specific promoter, resulted in glomerular-specific expression, with no extra-renal expression,^28^ giving us confidence that off-target expression can be mitigated.

In summary, this study suggests that *VEGFC* gene therapy mitigates the deleterious effects of diabetes on the glomerular filtration barrier and represents a novel and highly promising treatment strategy for DKD in T1D.

## Materials and methods

Please refer to supplemental materials and methods.

## Data and code availability

All summary data are included within the manuscript. There are no large datasets. Raw data can be provided upon request.

## Acknowledgments

A.M.A. was funded by 10.13039/501100000274BHF PG/20/10187, 10.13039/501100000291Kidney Research UK RP_005_20221128, and 10.13039/100019415Elizabeth Blackwell Institute MRC Confidence in Concepts Award 2019/20. C.M. was funded by 10.13039/501100000361Diabetes UK (grant 19/0006037). H.S.-C. was funded by a 10.13039/100010269Wellcome Trust Partnership Award Fellowship 2023. M.G. was funded by MRC project grant MR/T031921/1 and 10.13039/501100000361Diabetes UK (grant 19/0006037). K.O. was funded by Diabetes UK (grant 19/0006037). R.R. was funded by the 10.13039/501100000274British Heart Foundation (grant PG/22/11121). W.Y.D. was funded by an 10.13039/100006662NIHR Clinical Lectureship (grant CL-2019-25-003). The authors gratefully acknowledge the Wolfson Bioimaging Facility and the Animal Services Unit for their support and assistance in this work, and Sevil Erarslan Catak for kindly donating tissue sections. This has previously been submitted in abstract form at the European Society for Microcirculation Conference (2023), UK Kidney Week (2023), and the Annual Conference of the British Microcirculation and Vascular Biology Society (2023).

## Author contributions

Data curation, A.M.A., C.M., H.S.-C., H.W., M.G., K.O., and R.R.; formal analysis, A.M.A., C.M., H.S.-C., H.W., M.G., K.O., and R.R.; investigation, A.M.A. and C.M.; writing – original draft, A.M.A.; writing – review & editing, C.M., H.S.-C., H.W., M.G., K.O., R.R., W.Y.D., G.I.W., S.C.S., and R.R.F.; methodology, W.Y.D.; resources, W.Y.D.; supervision, G.I.W., S.C.S., and R.R.F.; conceptualization, R.R.F.; funding acquisition, R.R.F.; project administration, R.R.F.

## Declaration of interests

The authors have nothing to declare.

## Supplemental information

Document S1. Figures S1–S5 and supplemental materials and methods

Document S2. Article plus supplemental information
