# Supplementary material for: Podocyte-directed VEGFC gene therapy prevents increased glomerular permeability and glycocalyx damage in experimental type 1 diabetes
Source: Mol Ther. 2025 Oct 6;34(1):117–22. doi: 10.1016/j.ymthe.2025.10.001 (PMC12925785; doi:10.1016/j.ymthe.2025.10.001)
Supplement: Document S1. Figures S1–S5 and supplemental materials and methods [file mmc1.pdf]

YMTHE, Volume *34*

Supplemental Information

Podocyte-directed *VEGFC* gene therapy prevents increased glomerular permeability and glycocalyx damage in experimental type 1 diabetes

Aldara Martin Alonso, Carl J. May, Holly Stowell-Connolly, Haijie Wu, Monica Gamez, Khadija Ourradi, Raina D. Ramnath, Wen Yi Ding, Gavin I. Welsh, Simon C. Satchell, and Rebecca R. Foster

ai **aii**

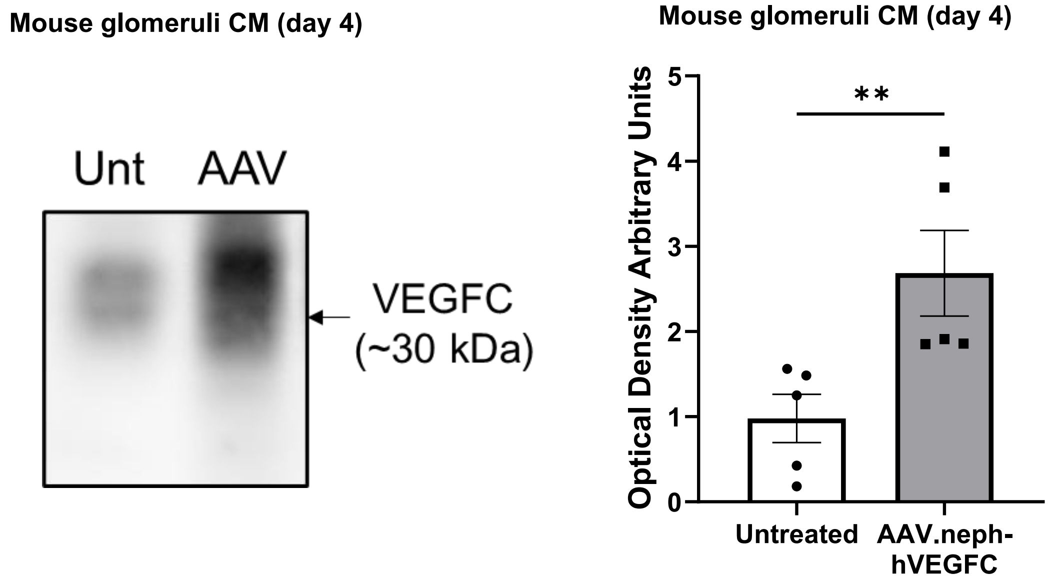

Figure S1 | VEGFC expression in conditioned media of mouse glomeruli at day 4 after *ex vivo* transduction with AAV.neph-*VEGFC*. Isolated murine glomeruli from wild type SV129 mice were transduced with AAV.neph-*VEGFC* (MOI 1 x 10^5; manufactured by VectorBuilder) and expression of hVEGFC was measured at day 4 after *ex vivo* infection by western blotting of the conditioned media (CM) (ai). Optical density arbitrary units (aii; Untreated, n = 5; AAV.neph-*VEGFC*, n = 5 (one-tailed unpaired t test)). All data are presented as mean ± SEM. $**p < 0.01$.

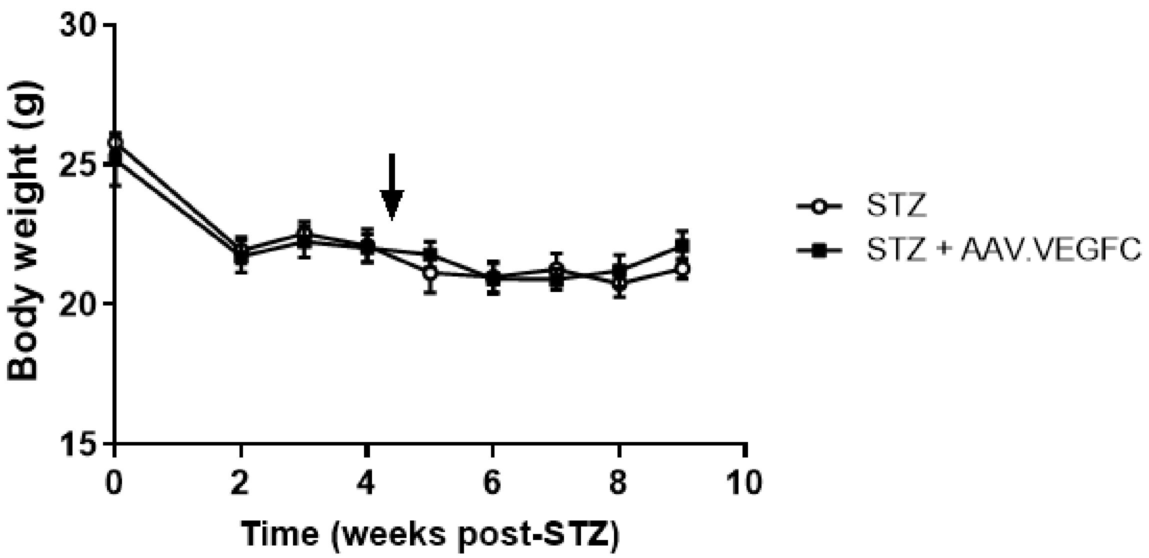

Figure S2 | Body weight of type 1 diabetic mice does not change with AAV.neph-*VEGFC* intervention. Body weight overtime post-STZ administration. The arrow indicates time of AAV.neph-*VEGFC* injection (AAV.*VEGFC*). STZ, n = 8 animals (n = 7 at week 9); STZ + AAV.neph-*VEGFC*, n = 9 animals. All data are presented as mean ± SEM.

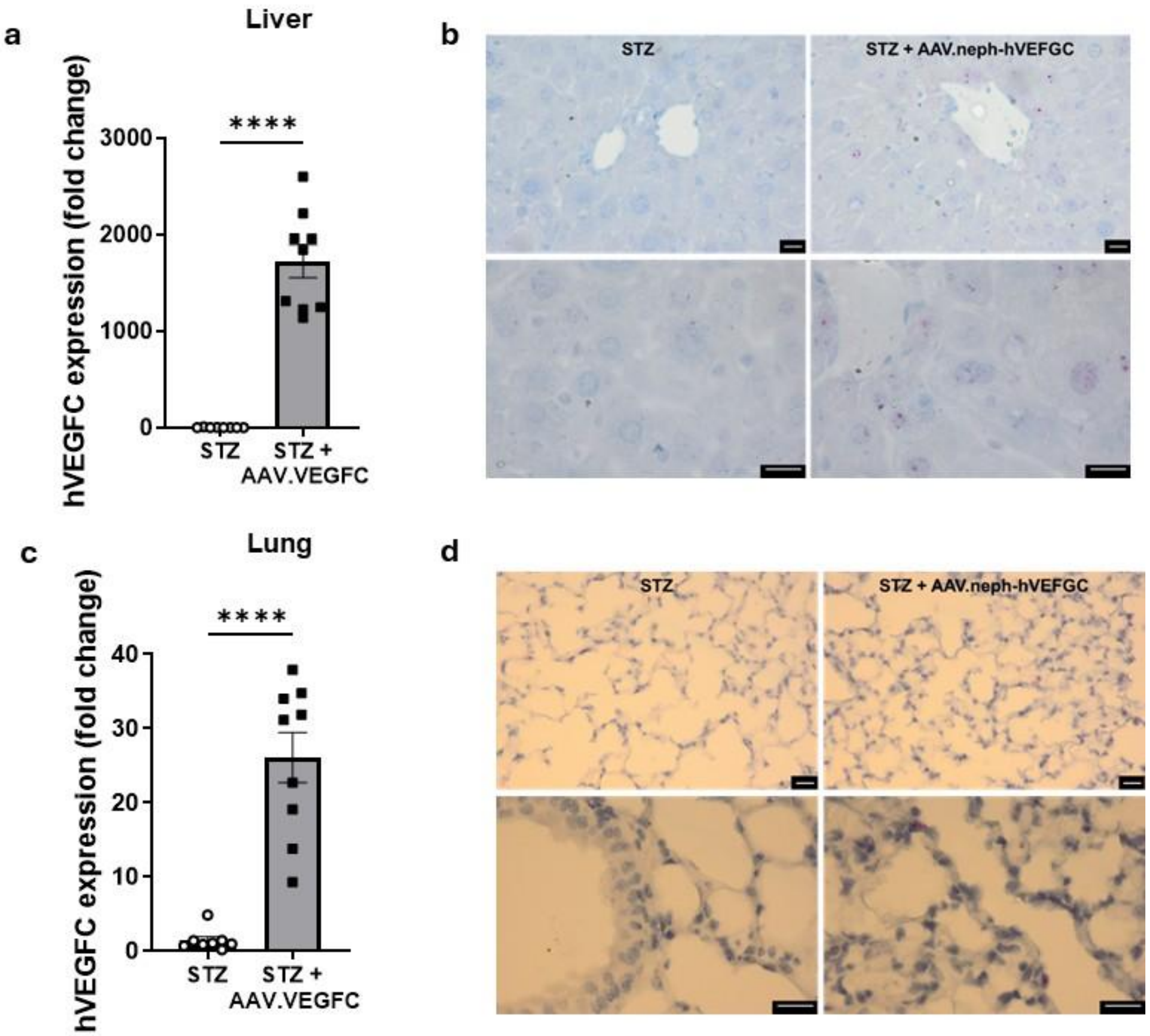

Figure S3 | Transgene expression in other tissues of type 1 diabetic mice treated with AAV.neph-*VEGFC*. a) Human VEGFC mRNA expression in liver tissue. STZ, n = 8 animals; STZ + AAV.neph-*VEGFC*, n = 9 animals. One-tailed unpaired t test. b) Representative images of chromogenic (red) in situ hybridization of WPRE in liver sections. Magnifications 20x (top) and 40x (bottom). Scale bar: 20 µm. c) Human VEGFC mRNA expression in lung tissue. STZ, n = 8 animals; STZ + AAV.neph-*VEGFC*, n = 9 animals. One-tailed unpaired t test. d) Representative images of chromogenic (red) in situ hybridization of WPRE in lung sections. Magnifications 20x (top) and 40x (bottom). Scale bar: 20 µm. ****P < 0.0001. All data are presented as mean ± SEM.

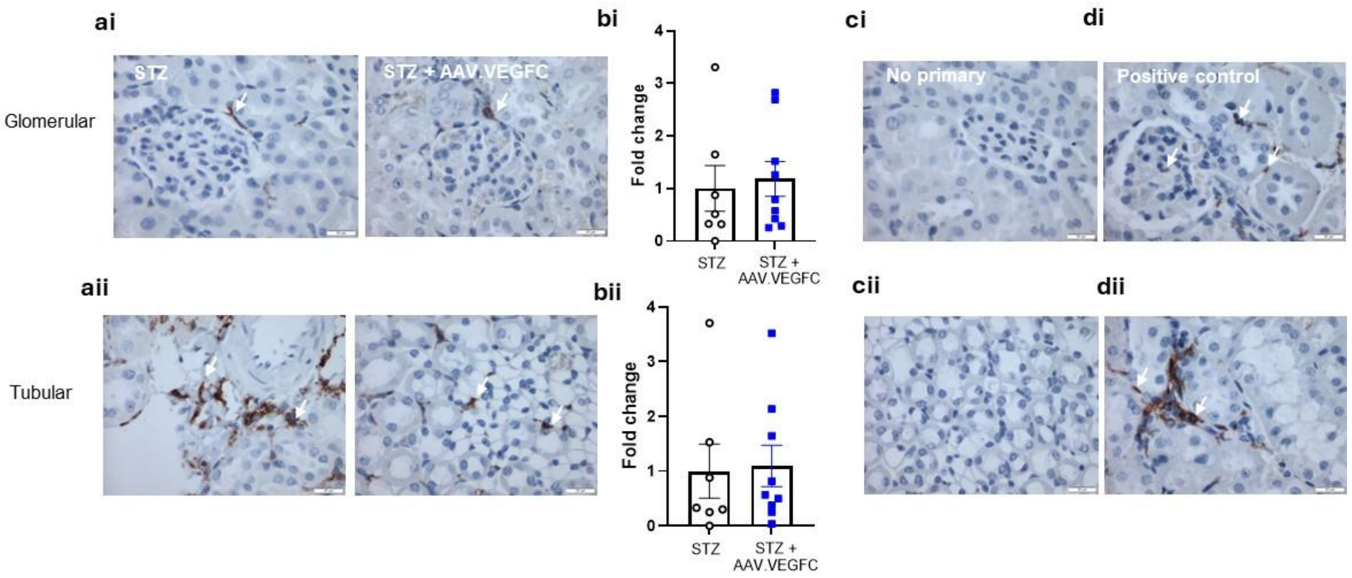

Figure S4 | AAV.neph-*VEGFC* does not impact on glomerular or tubular macrophage staining. (a) Representative images of F4/80 immunohistochemistry staining in glomerular (ai) and tubular (aii) areas of the kidney from STZ (n = 8) and STZ + AAV.neph-*VEGFC* (AAV.*VEGFC*) (n = 9) mice. (b) Quantification of F4/80 staining using integrated density and normalised to the STZ group for glomerular (bi) and tubular (bii) staining. (c) Representative images showing no primary antibody negative control in glomerular (ci) and tubular (cii) regions. (d) Representative images of tissue sections from a Type 2 mouse model of diabetes at 16 wk for glomerular (di) and tubular (dii) staining. White arrows indicate positive staining. Scale bar: 20 µm. All data are presented as mean ± SEM.

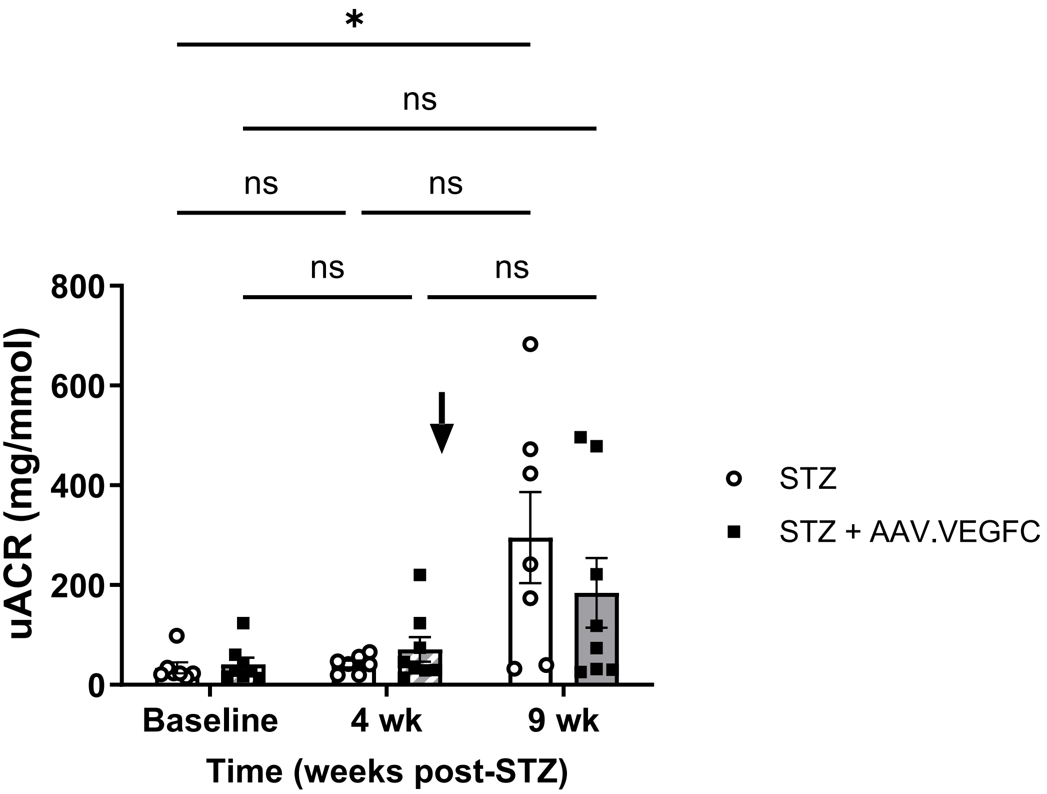

Figure S5 | Urine albumin-to-creatinine ratio of type 1 diabetic mice treated with AAV.neph-*hVEGFC*. Absolute uACR after injection of AAV.neph-*VEGFC* (arrow). Two-way ANOVA followed by Dunnett's multiple comparison test. STZ, n = 7 animals; STZ + AAV.neph-*hVEGFC*, n = 8 animals. *P < 0.05. All data are presented as mean ± SEM.

Supplementary Materials and Methods

Virus production

Initially, the AAV particles used were produced in house. The Flag-tagged human *VEGFC* gene (NCBI Ref Seq: NM_005429.2, Sino Biologicals) was cloned into an AAV9 plasmid, which contains the minimal human nephrin promoter (provided by Dr Wen Ding) and AAV2 ITRs, using *AflII* and *SbfI* restriction sites. Viral particles were produced in HEK293T by triple transfection with the AAV plasmid (the helper plasmid with adenoviral genes (pHGTI-Adeno1) and the capsid plasmid as previously described.[1] The capsid AAV9 sequence was used to target mouse podocytes.[1] The AAV preparation was purified by iodixanol gradient ultra-centrifugation using a Type70.1 Ti rotor, concentrated in phosphate-buffered saline (PBS) and titrated by alkaline gel electrophoresis and qPCR targeting the ITR sequence. Later, AAV manufacturing was outsourced (VectorBuilder).

Cell culture

Podocyte and glomerular endothelial cell lines used in this work are conditionally immortalised cell lines generated in house and cultured as previously described.[2-4] These cell lines were immortalised with a SV40 temperature-sensitive T antigen. Cell lines proliferate at 33°C and, when switched to 37°C, they become differentiated. Both human and mouse podocytes were cultured with RPMI-1640 containing L-glutamine and $NaHCO_3$ supplemented with 10% FBS. Human conditionally immortalised glomerular endothelial cells (GEnC) were cultured with the EBM-2 Endothelial Cell Growth Basal Medium-2 supplemented with EGM-2 Endothelial Cell Growth Medium-2 BulletKit (Lonza), except for the GA-1000. Conditionally immortalised podocytes at a confluence of 30-50% were transduced with AAV and thermoswitched to 37°C. For VEGFC expression, podocytes were used 10-20 days after transduction, when cells are fully differentiated.

RNA extraction and quantitative PCR

RNA was extracted using Tri reagent (Sigma Aldrich) and treated with RQ1 DNAse kit (Promega) following supplier instructions. cDNA was synthesized using a High-Capacity RNA-

to-cDNA kit (Thermo Fisher Scientific). Quantitative qPCR was performed in triplicates using TaqMan Fast Advanced Master Mix (Thermo Fisher Scientific) and TaqMan Gene Expression Assays (Thermo Fisher Scientific; Human VEGFC: Hs00153458_m1; Mouse GAPDH: Mm99999915_g1; Human GAPDH: Hs02786624_g1) following supplier instructions in a StepOnePlus Real-Time PCR System (Applied Biosystems). The fold change was calculated using the $2^{-\Delta\Delta Ct}$ method.

Western blotting

Protein lysates were subjected to SDS-PAGE under reducing conditions and transferred to a PVDF membrane. Membranes were blocked and incubated with primary antibodies overnight at 4°C (anti-human VEGFC (Cat. MA5-26494, Thermo Fisher Scientific) at 1:200; anti-pan phosphotyrosine antibody (cat. SAB5700563, Sigma-Aldrich) at 1:1000; anti-VEGFR2 (cat. AF357, R&D) at 1:1000) before washing and incubation with HRP-conjugated secondary antibodies. After adding the Clarity ECL Western Blotting substrate (Bio-Rad), bands were visualised in an Amersham Imager 600 (GE Healthcare Life Sciences) and quantified in ImageJ (NIH).

Immunoprecipitation

Immunoprecipitation was performed using the Thermo Scientific Pierce™ Classic IP kit (cat. 26146) and the anti-VEGFR2 (cat. 55B11, Cell Signalling Technologies). Then samples were subjected to western blotting as described above.

Primary glomeruli culture

Glomeruli were sieved from kidney cortex as previously described.[3] Human glomeruli were isolated from kidneys that were unsuitable for transplantation. Use of human kidney tissue was approved by national and local research ethics committees and conducted in accordance with the tenets of the Declaration of Helsinki. For mouse glomeruli, a yield of ~8000 glomeruli was obtained from two adult mouse kidneys. Glomeruli were cultured in supplemented EBM-2 medium.

Animals

All animal experiments and procedures were approved by the UK Home Office and approved locally by AWERB committee. In this study, *n* refers to the number of animals. Animals were maintained in a controlled environment (21-24°C and 12:12 h light-dark cycle). Animals were acclimatised for 1 week after arrival. Standard laboratory chow and drinking water were provided *ad libitum* (unless otherwise required for experimental reasons). We carried out an initial pilot study of 6 wk old male wild type C57BL/6 mice, given $1.5x10^{12}$ AAV.*VEGFC* by tail vein injection or left untreated (n=3 each group). Urine was collected weekly for 5 wk. Glomerular hVEGFC mRNA was confirmed in n=2 animals (13.5 ± 7.4 fold increase). Mice were macroscopically normal and uACR remained unchanged (untreated: 11.1 ± 5.8 AAV.*VEGFC*: 11.9 ± 3.2 mg/mmol), confirming that AAV.*VEGFC* could be tolerated well and was not harmful. In this study, we used a model of early diabetic kidney disease.[5] Diabetes was induced in a total of 18 10-week-old DBA2/J male mice (Charles River) by intraperitoneal injection of streptozotocin (STZ) following the low-dose STZ protocol (50 mg/kg over 5 consecutive days) from the *Diabetic Complications Consortium*. Blood glucose was measured by tail-tip blood droplet analysis using a glucometer (GlucoRx Nexus). Body weight was regularly monitored. STZ-injected mice were considered diabetic when blood glucose levels were ≥ 15 mmol/l. Urine was collected by placing mice in cages containing LabSand (Braintree Scientific, Inc.) for up to 3 hours. Four weeks after STZ administration, a subgroup (n=9) mice received AAV-*VEGFC* particles (custom made by VectorBuilder) via tail vein injection at a dose of $7.50x10^{13}$ GC/kg. The random allocation of mice to the AAV treatment group was done using the RAND function in Microsoft Excel while ensuring groups were balanced per cage and that body weight and blood glucose were balance between groups. The sample size accounted for the success rate and mortality rates of the method used to induce albuminuria (primary outcome) and on success rate of tail vein injection. Not all animals were used for all outcomes because of the diabetes adverse effects. Humane endpoints were animals showing overt signs of ill-health and/ or weight loss of >20% from baseline, and which did not improve in response to supportive measures (hydration, heat pad). Two animals were terminated by schedule 1 due to diabetic complications at endpoint. Mice were terminated 9 or 10 weeks after STZ

administration. Tissue was collected and either snap frozen in liquid nitrogen, or fixed. Some mice were cardiac perfused with Ringer solution (NaCl, 132mM; KCl, 4.6mM; $MgSO_4$-$7H_2O$ 1.27mM; $CaCl_2$-$2H_2O$ 2mM; $NaHCO_3$, 25mM; D(+)glucose, 5.5mM; N-2-hydroxyethylpiperazine-N'-2-ethanesulphonic (HEPES) acid, 3.07mM; HEPES sodium salt, 1.9mM, pH 7.40) before termination and then tissue collected. Glomeruli were isolated from perfused kidney pieces by graded sieving and collected from the 75 µm pore sieve. Then, glomeruli were either snap frozen or used in the glomerular albumin permeability assay. The investigator was aware of the treatment groups during the experiment but blinded for data analysis.

Urinary albumin-to-creatinine ratio (uACR)

Urinary albumin was quantified using a mouse albumin ELISA (Bethyl Laboratories Inc.) following manufacturer's instructions. Creatinine was measured by the Bristol Langford Clinical Veterinary Service. If uACR was abnormally high, animals were excluded from urinary albumin-creatinine ratio and glomerular albumin permeability (Ps'alb) assay data analysis.

Glomerular albumin permeability assay

The glomerular albumin permeability (Ps'alb) assay was performed as previously described.[6] Briefly, after cardiac perfusing animals with Ringer buffer, kidneys were isolated by graded sieving with 4% BSA in Ringer solution. Glomeruli were incubated with 36.5 µg/ml of R18 (Thermo Fisher Scientific) for 15 min on ice, washed twice in BSA/Ringer and incubated with 30 µg/mL Alexa Fluor 488-BSA (Thermo Fisher Scientific) for 15 min on ice. An individual glomerulus was trapped on a custom-made dish under a Nikon Ti-E inverted confocal microscope (Nikon Instruments, Inc.). While imaging, the perfusate was switched from Alexa Fluor 488-BSA to unlabelled BSA. The rate of decrease in fluorescence intensity within the loop of the capillaries was quantified blinded using the NIS Elements Analysis software (Nikon Instruments, Inc.) and used to calculate Ps'alb.

***In situ* hybridization**

Tissue was fixed in 10% neutral buffered formalin for 16-32 hours at room temperature, paraffin-embedded and sectioned. Sections were probed for Woodchuck Hepatitis Virus

Posttranscriptional Regulatory Element (WPRE) using the RNAscope® 2.5 High-Definition Red Assay (ACD-Biotechne). The manufacturer's instructions were followed with some changes. All sections were subjected to a 30-minute incubation in 0.3M HCl following the deparaffinisation steps. Target retrieval duration was 15 min in kidney and lung sections, while 30 min in liver sections. All sections were incubated in Protease Plus for 30 minutes at 40°C. After incubation with RNAscope® probe WPRE-O2 (2 hrs at 40°C), sections were kept in SSC buffer overnight and the assay continued the following day. Incubation in AMP5 was 90 min at room temperature.

Imaging was performed in Leica DMI6000 widefield microscope with motorised stage and DFC420C camera using the Leica LASX acquisition software or in a Leica DMLB microscope with an Olympus Colorview I camera and Olympus CellSens Entry v1.15 software.

For quantification in kidney sections, images of glomeruli were randomly obtained by acquiring tile scans and the ImageJ (NIH) PlugIn 'Colour Deconvolution' was used. A threshold was set to define positive-WPRE and background staining, respectively, and images were converted to a binary mask. Staining area in the glomerulus was measured by defining a region of interest using the original images, and the mean WPRE staining was normalised to the mean background stain.

Lectin staining

Paraffin-embedded kidney sections (3 µm) were dewaxed in xylene for 10 mins followed by rehydration in graded ethanol and washing in distilled water. After three washes with PBS-tween 0.1% (pH 7.4), sections were incubated with 1% BSA in PBS-tween 0.1% (pH 7.4) for 1 h and wash again. Sections were incubated with FITC-labelled *Lycopersicon esculentum* lectin (LEL-FITC; Sigma-Aldrich) at 1:100 in 1% BSA in PBS-tween 0.5% (pH 6.8) overnight at 4°C. After washing twice with PBS-tween 0.1%, sections were incubated with 300 nM DAPI (Sigma-Aldrich) for 10 min. After washing twice with PBS-tween 0.1% (pH 7.4), sections were incubated for 15 min with Octadecyl Rhodamine B Chloride (R18; Thermo Fisher Scientific) diluted 1:1000 in PBS. After dipping the slides 5 times in PBS, coverslips were mounted using

Vectashield (Vector Laboratories). Sections were imaged using a Leica SP8 AOBS confocal laser scanning microscope attached to a Leica DM I8 inverted epifluorescence microscope.

Endothelial glycocalyx depth analysis

The distance between the peak signals from the LEL-FITC and R18 fluorophores (peak-to-peak) is an index of glycocalyx depth. Peak-to-peak measurements were performed blinded using an ImageJ (NIH) macro.[7] The mean was determined from 360 lines per capillary loop, 1-4 loops per glomerulus, and 4-7 glomeruli per mouse.

Macrophage staining and quantification

Fresh kidney cortex was fixed in 4% PFA overnight and stored in 70% ethanol. Tissues were embedded in paraffin and 5 µm sections were cut. Sections were dewaxed with xylene and hydrated with ethanol (100%, 90% and 70%) and distilled water. Antigen retrieval was performed using 10 mM sodium citrate tribasic buffer (pH 6.0), followed by inactivation of endogenous peroxidase with hydrogen peroxide (3 wt.%, 88597, Merck, Darmstadt, Germany). After blocking with 5% normal goat serum (ab7481, Abcam, Cambridge, UK) in TBST, the sections were incubated with F4/80 (1:400, 70076T, Cell Signalling Technology, Leiden, Netherlands) overnight at 4 °C. After 3 washes with TBST, sections were incubated with HRP reagent (SignalStain® Boost Detection Reagent, 8114P, Cell Signalling Technology, Leiden, Netherlands) for 30 minutes at room temperature. The positive staining was apparent with SignalStain® DAB Substrate Kit (8059P, Cell Signalling Technology, Leiden, Netherlands). Sections were counterstained with hematoxylin for 10 seconds and dehydrated with ethanol and xylene. DPX Mountant (06522, Sigma-Aldrich, Gillingham, UK) was used to seal the sections with coverslips. Images were taken using a Leica DMLB Microscope (Leica Microsystems, Milton Keynes, UK) with an Olympus Colorview I camera and Olympus cellSens Entry v1.15 software. Macrophage staining was quantified by mean % positive stained area in glomerular and tubular regions via ImageJ.

Statistical analysis

Data are expressed as mean ± SEM. Prism 10 software (GrapPad Software, LLC) was used for analysis. Normality tests were performed. Statistical tests used to evaluate changes are

indicated in figure legends. A P value of < 0.05 was considered to indicate a significant difference.

References

1. Ding, WY, Kuzmuk, V, Hunter, S, Lay, A, Hayes, B, Beesley, M, Rollason, R, Hurcombe, JA, Barrington, F, Masson, C, Cathery, W, May, C, Tuffin, J, Roberts, T, Mollet, G, Chu, CJ, McIntosh, J, Coward, RJ, Antignac, C, Nathwani, A, Welsh, GI, Saleem, MA: Adeno-associated virus gene therapy prevents progression of kidney disease in genetic models of nephrotic syndrome. *Sci Transl Med,* 15**:** eabc8226, 2023.
2. Satchell, SC, Tasman, CH, Singh, A, Ni, L, Geelen, J, von Ruhland, CJ, O'Hare, MJ, Saleem, MA, van den Heuvel, LP, Mathieson, PW: Conditionally immortalized human glomerular endothelial cells expressing fenestrations in response to VEGF. *Kidney Int,* 69**:** 1633-1640, 2006.
3. Saleem, MA, O'Hare, MJ, Reiser, J, Coward, RJ, Inward, CD, Farren, T, Xing, CY, Ni, L, Mathieson, PW, Mundel, P: A conditionally immortalized human podocyte cell line demonstrating nephrin and podocin expression. *J Am Soc Nephrol,* 13**:** 630-638, 2002.
4. Keir, LS, Firth, R, May, C, Ni, L, Welsh, GI, Saleem, MA: Generating conditionally immortalised podocyte cell lines from wild-type mice. *Nephron,* 129**:** 128-136, 2015.
5. Onions, KL, Gamez, M, Buckner, NR, Baker, SL, Betteridge, KB, Desideri, S, Dallyn, BP, Ramnath, RD, Neal, CR, Farmer, LK, Mathieson, PW, Gnudi, L, Alitalo, K, Bates, DO, Salmon, AHJ, Welsh, GI, Satchell, SC, Foster, RR: VEGFC Reduces Glomerular Albumin Permeability and Protects Against Alterations in VEGF Receptor Expression in Diabetic Nephropathy. *Diabetes,* 68**:** 172-187, 2019.
6. Desideri, S, Onions, KL, Qiu, Y, Ramnath, RD, Butler, MJ, Neal, CR, King, MLR, Salmon, AE, Saleem, MA, Welsh, GI, Michel, CC, Satchell, SC, Salmon, AHJ, Foster, RR: A novel assay provides sensitive measurement of physiologically relevant changes in albumin permeability in isolated human and rodent glomeruli. *Kidney Int,* 93**:** 1086-1097, 2018.
7. Crompton, M, Ferguson, JK, Ramnath, R, Onions, KL, Ogier, AS, Gamez, M, Down, CJ, Skinner, LJ, Wong, KH, Dixon, LK, Sutak, J, Harper, SJ, Pontrelli, P, Gesualdo, L, Heerspink, HL, Toto, RD, Welsh, GI, Foster, RR, Satchell, SC, Butler, MJ: Mineralocorticoid receptor antagonism in diabetes reduces albuminuria by preserving the glomerular endothelial glycocalyx. *JCI insight*, 2023.
