# Supplementary material for: Podocyte-directed VEGFC gene therapy prevents increased glomerular permeability and glycocalyx damage in experimental type 1 diabetes
Source: Mol Ther. 2025 Oct 6;34(1):117–22. doi: 10.1016/j.ymthe.2025.10.001 (PMC12925785; doi:10.1016/j.ymthe.2025.10.001)
Supplement: Document S2. Article plus supplemental information [file mmc2.pdf]

Molecular Therapy
Brief report

Podocyte-directed *VEGFC* gene therapy prevents increased glomerular permeability and glycocalyx damage in experimental type 1 diabetes

Aldara Martin Alonso,[1] Carl J. May,[1] Holly Stowell-Connolly,[1] Haijie Wu,[1] Monica Gamez,[1] Khadija Ourradi,[1] Raina D. Ramnath,[1] Wen Yi Ding,[1] Gavin I. Welsh,[1] Simon C. Satchell,[1] and Rebecca R. Foster[1]

[1]Bristol Renal, Translational Health Sciences, Bristol Medical School, University of Bristol, Bristol BS1 3NY, UK

Diabetic kidney disease (DKD) is the leading cause of end-stage renal failure, and current interventions fail to directly target the glomerulus, where the disease initiates. Vascular endothelial growth factor (VEGF)C is a key contributor to glomerular endothelial barrier function. In transgenic mice, podocyte-specific overexpression of human VEGFC was protective in early DKD. Here, we investigated the therapeutic potential of a podocyte-targeted *VEGFC* gene therapy in DKD. We employed an adeno-associated virus (AAV2/9) to drive human VEGFC in human and mouse podocytes. Expressed VEGFC was functional *in vitro*. In type 1 diabetic mice (induced by streptozotocin), systemic administration of AAV2/9 increased glomerular human VEGFC expression, ameliorating both albuminuria and increased glomerular permeability. Importantly, *VEGFC* gene therapy also protected the glomerular endothelial glycocalyx, the first barrier to protein in the glomerular filtration barrier. These findings demonstrate that podocyte-directed *VEGFC* gene delivery can restore glomerular function and protect against early DKD progression. This novel approach represents a promising therapeutic strategy, particularly for patients with type 1 diabetes at risk of DKD, where there is an unmet clinical need.

INTRODUCTION

Diabetic kidney disease (DKD) is the most prevalent long-term complication of diabetes and the leading cause of end-stage renal disease in the Western world. The incidence of DKD is expected to rise, as global diabetes cases have quadrupled globally since 1990 and could more than double by 2050.[1] In type 1 diabetes (T1D), up to 30% of these individuals develop DKD within 10 years of diagnosis.[2] The UK was ranked fifth globally for T1D incidence among children aged 0–14 years, with a rate of 25 cases per 100,000 children, in 2021 (NHS England). Once DKD progresses, kidney damage is irreversible, limiting treatment options to dialysis or a kidney transplant. These treatments not only diminish patients' quality of life, but also impose a substantial economic burden. DKD accounts for 21% of deaths in people with T1D and 11% in those with type 2 diabetes (T2D).[3] Recent renoprotective therapeutic advances for T2D have not been approved for T1D, highlighting a significant unmet clinical need in patients with T1D at risk of DKD.

Increasing albuminuria is the earliest indicator of DKD, reflecting damage to the glomerular endothelium before ultrastructural changes in podocytes are observed.[4] Diabetes induces glomerular endothelium dysfunction, particularly by damaging the proteoglycan-rich layer coating the glomerular endothelium, known as the endothelial glycocalyx (eGlx). Despite this, glomerular endothelial-specific therapies for DKD have not been developed. Glomerular vascular endothelial growth factor (VEGF)C, expressed by podocytes, enhances the glycocalyx barrier properties of the glomerular endothelium.[5–7] Using a transgenic mouse model, we have demonstrated the therapeutic potential of podocyte-specific human VEGFC overexpression in experimental DKD.[7] Herein, we take the next step toward translation, hypothesizing that *VEGFC* gene therapy will ameliorate albuminuria in a T1D model. This advanced approach employs an adeno-associated virus (AAV)-based gene therapy tool that can effectively target podocytes.[8]

RESULTS

AAV induces the expression of functional human VEGFC in podocytes *in vitro*/*ex vivo* using a minimal nephrin promoter

AAV.neph-*VEGFC* was initially manufactured in-house. Mouse conditionally immortalized podocytes were transduced with AAV.neph-*VEGFC* particles to confirm *VEGFC* expression. *VEGFC* mRNA expression, quantified by RT-qPCR, significantly increased in a dose-dependent manner in podocytes transduced at different multiplicities of infection (MOIs) of the AAV (Figure 1A). hVEGFC was measured in the conditioned media of AAV-transduced mouse podocytes by western blotting, using media from HEK (human embryonic kidney) cells previously transfected with a *VEGFC*-expressing plasmid as positive controls (Figure 1Bi). Secreted hVEGFC expression was significantly higher in AAV-transduced podocytes compared

Received 14 July 2025; accepted 2 October 2025;
https://doi.org/10.1016/j.ymthe.2025.10.001.

Correspondence: Rebecca Foster, Bristol Renal, Translational Health Sciences, Bristol Medical School, University of Bristol, Bristol BS1 3NY, UK.
E-mail: becky.foster@bristol.ac.uk

© 2025 The Authors.
Published by Elsevier Inc. on behalf of The American Society of Gene and Cell Therapy.
This is an open access article under the CC BY license (http://creativecommons.org/licenses/by/4.0/).

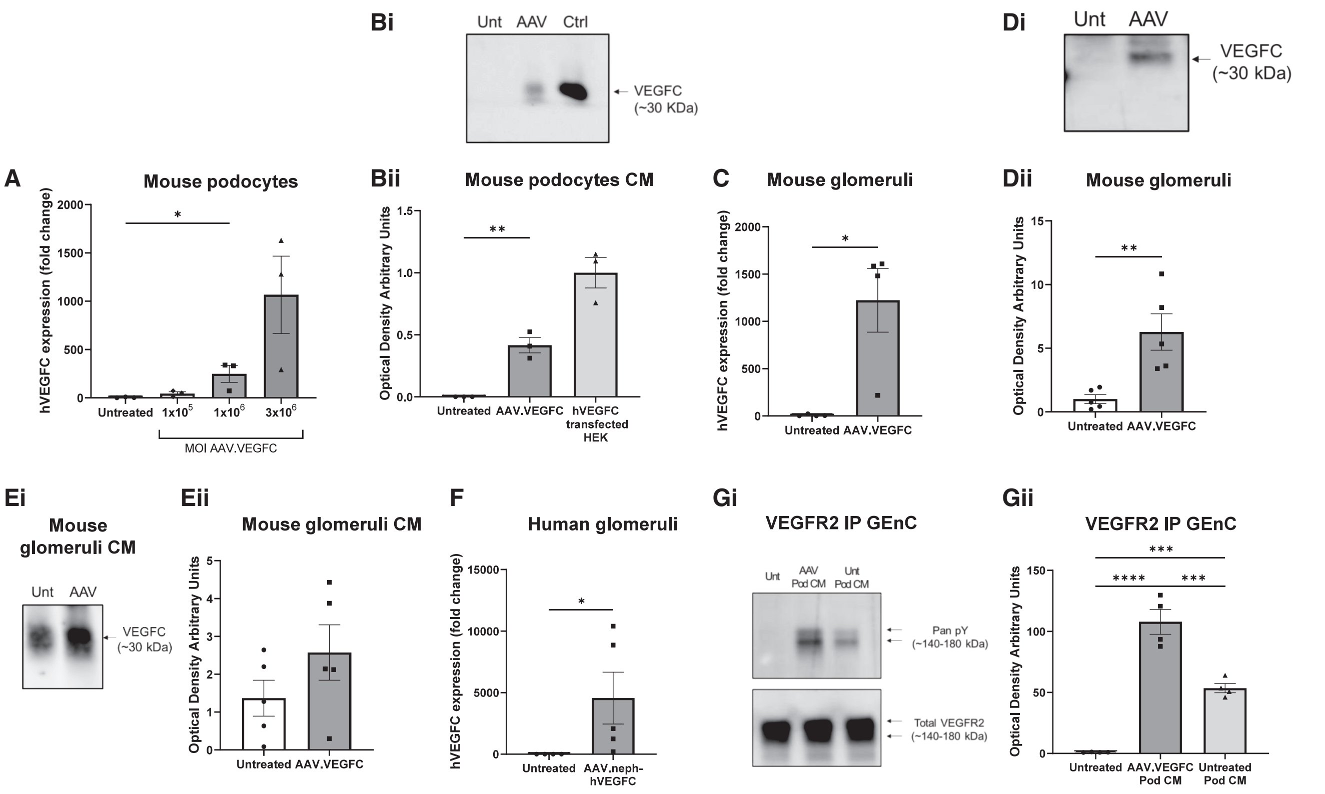

Figure 1. AAV induces the expression of functional human VEGFC in podocytes *in vitro*/*ex vivo* using a minimal nephrin promoter

Conditionally immortalized mouse podocytes (Pods) were infected with AAV.neph-*VEGFC* (AAV.*VEGFC*) (manufactured in-house) at different multiplicity of infection (MOI) doses, and transduction of human *VEGFC* mRNA was shown by qPCR (A). Expression is relative to mouse GAPDH and untreated (Unt) control ($2^{-\Delta\Delta Ct}$). Unt, $n = 3$; AAV.*VEGFC*, $n = 3$ (Kruskal-Wallis test, $p < 0.01$, followed by Dunn's multiple comparison test of Unt vs. MOI 1×10^6). (B) Detection of human VEGFC protein in concentrated conditioned medium (CM) from mouse Pods transduced with AAV.*VEGFC* (MOI 1×10^6; manufactured in-house) by western blotting. HEK293T cells were transfected with a plasmid driving *VEGFC* expression under the cytomegalovirus (CMV) promoter as a positive control of the blot. A representative blot is shown (Bi), and data are summarized (Bii). Unt, $n = 3$; AAV.*VEGFC* (AAV), $n = 3$; hVEGFC-transfected HEK (Ctrl), $n = 3$ (one-way ANOVA, $p < 0.001$, followed by Šídák's multiple comparisons test of Unt vs. AAV-transduced podocyte CM). Isolated murine glomeruli from wild-type SV129 mice were transduced with AAV.neph-*VEGFC* (MOI 1×10^5; manufactured by VectorBuilder), and expression of *VEGFC* was measured on day 5 after *ex vivo* infection by qPCR (C; Unt, $n = 4$; AAV.neph-*VEGFC*, $n = 4$; one-tailed Mann-Whitney test), by western blotting of the glomeruli; a representative blot is shown (Di), and data are summarized (Dii) (Unt, $n = 5$; AAV.neph-*VEGFC*, $n = 5$ [unpaired *t* test]), and of the CM; a representative blot is shown (Ei), and data are summarized (Eii) (Unt, $n = 5$; AAV.neph-*VEGFC*, $n = 5$ [unpaired *t* test]). (F) hVEGFC qPCR on day 5 post-*ex-vivo* transduction of isolated human glomeruli with AAV.*VEGFC* (MOI 1×10^5; ultrapure particles manufactured by VectorBuilder). Unt, $n = 4$; AAV.*VEGFC*, $n = 5$ (unpaired *t* test). (Gi) Immunoprecipitation (IP) with anti-VEGFR2 antibody of conditionally immortalized human glomerular endothelial cells (GEnCs) treated for 15 min with CM from mouse Pods previously transduced with AAV.*VEGFC* (manufactured in-house) or Unt (10 days) and western blotting with a pan anti-phosphotyrosine antibody (pan pY) or anti-VEGFR2. Unt GEnC (Unt), $n = 4$; AAV.*VEGFC* Pod CM (AAV Pod CM), $n = 4$; Unt Pod CM (Unt Pod CM), $n = 4$. (Gii) Optical density normalized to total VEGFR2 and relative to Unt GEnC (one-way ANOVA followed by Tukey's multiple comparisons test, $p < 0.0001$). All data are presented as mean ± SEM. *$p < 0.05$, **$p < 0.01$, ***$p < 0.001$, and ****$p < 0.0001$.

to untreated podocytes (Figure 1Bii). Isolated wild-type mouse glomeruli were transduced with AAV.neph-*VEGFC* particles. At day 5 post-AAV transduction, mouse glomeruli showed a significant increase in *VEGFC* mRNA (Figure 1C) and protein expression by western blotting (Figures 1Di and 1Dii) and a trend toward increased VEGFC secretion, as shown by western blotting of the conditioned media (Figures 1Ei and 1Eii) that peaked at day 4 (Figure S1). Importantly, AAV-transduced isolated human glomeruli also showed a significant increase in *VEGFC* mRNA expression (Figure 1F). Thus, we have demonstrated successful gene delivery to *ex vivo* human glomeruli and demonstrated that the AAV2/9 serotype was effective for both mouse and human tissue.

To demonstrate the functionality of AAV-induced hVEGFC, human conditionally immortalized glomerular endothelial cells (GEnCs) were treated with conditioned media from AAV-transduced mouse podocytes, and activation of the endothelial-specific receptor tyrosine kinase VEGFR receptor 2 (VEGFR2) was assessed. This activation is crucial for mediating VEGFC's effects on GEnC barrier properties.[5,7] GEnC protein was immunoprecipitated using an anti-VEGFR2 antibody, and then phosphorylated tyrosine (pY) and total VEGFR2 were detected by western blotting (Figure 1Gi). Conditioned media from AAV-treated podocytes significantly increased pY in GEnCs at the same molecular weight as VEGFR2, indicating that secreted hVEGFC triggers VEGFR2 phosphorylation in GEnC (Figure 1Gii).

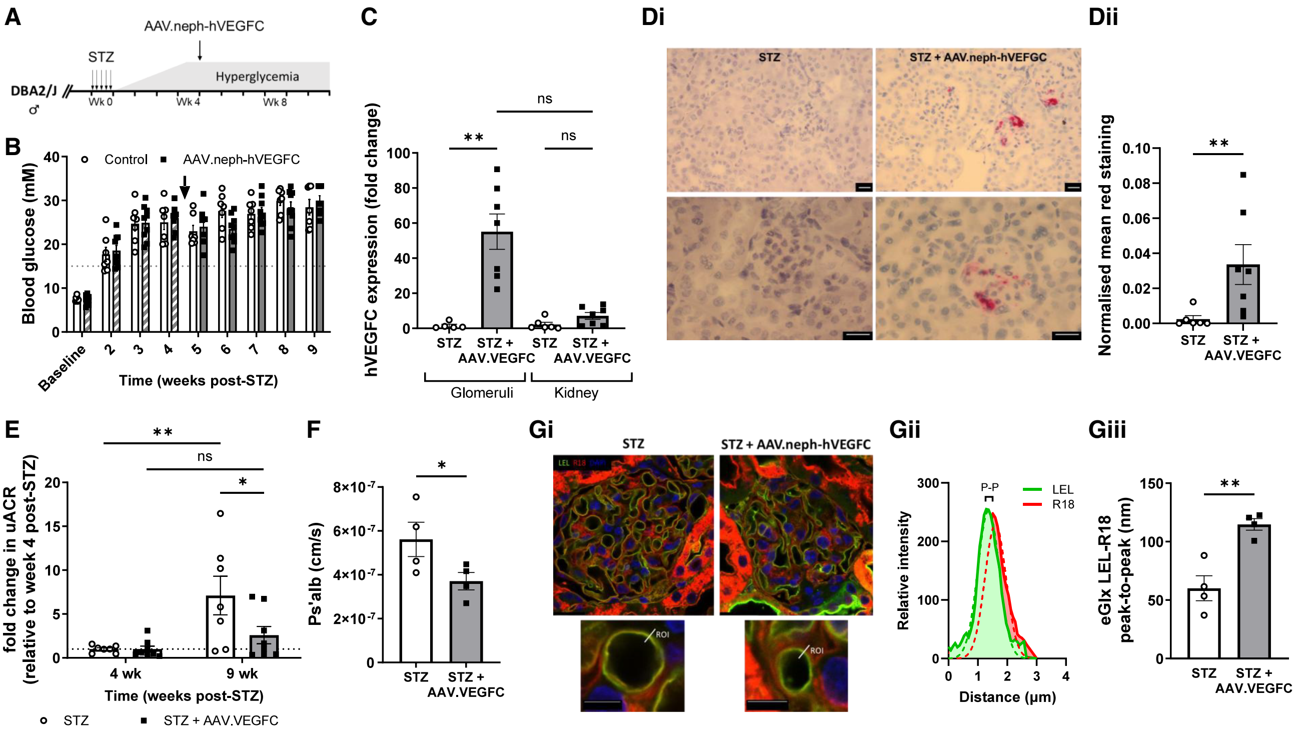

Figure 2. AAV.neph-*VEGFC* ameliorates albuminuria and restores the glomerular endothelial glycocalyx in type 1 diabetes

DBA2/J male mice were given streptozotocin (STZ) during week 0 and then AAV.neph-*VEGFC* (AAV.*VEGFC*) (ultrapure particles manufactured by VectorBuilder) by tail vein injection (dose: 7.50 × 10^{13} GC/kg of body weight) at week 4 (A). Mice were terminated at week 9–10 post-STZ administration. (B) Weekly blood glucose (if out of range [>33.3 mM], it was plotted as 33.3 mM). Grid line: 15 mM threshold. Arrow: virus injection 4 weeks following STZ induction. STZ, $n = 7$; STZ + AAV.*VEGFC*, $n = 9$ (two-way ANOVA, $p < 0.0001$). (C) *VEGFC* qPCR of isolated glomeruli and kidney (week 9–10 post-STZ). Expression is relative to mouse GAPDH and control mice ($2^{-\Delta\Delta Ct}$). STZ, n = 5–6; STZ + AAV.*VEGFC*, $n = 7$. Kruskal-Wallis test ($p < 0.001$) followed by Dunn's multiple comparison tests. (Di) Kidney sections were subjected to chromogenic (red) *in situ* hybridization of WPRE (under the control of a nephrin promoter, such as *VEGFC*). Magnifications: 20× (top) and 40× (bottom). Scale bar: 20 μm. (Dii) Glomerular red signal normalized to background. STZ, $n = 6$ animals; STZ + AAV.*VEGFC*, $n = 7$ animals (Mann-Whitney one-tailed test). (E) Urine albumin-to-creatinine ratio (uACR) relative to uACR at week 4 post-STZ (just before virus injection). STZ, $n = 7$; STZ + AAV.*VEGFC*, $n = 8$ (two-way ANOVA followed by Bonferroni's multiple comparison test to compare animal groups or Šídák's multiple comparisons test to compare time points within each animal group). Absolute uACR in Figure S2. (F) In cardiac perfused mice, sieved glomeruli were loaded with octadecyl rhodamine B chloride (R18) and Alexa Fluor 488-BSA and subjected to perfusion with unlabeled BSA to measure albumin permeability (Ps'alb). One-tailed unpaired *t* test ($p < 0.05$) of the average Ps'alb of animals (STZ [$n = 4$], $n = 21$ glomeruli; STZ + AAV.*VEGFC* [$n = 4$], $n = 26$ glomeruli). We have previously reported that DBA2/J male mice receiving vehicle (citrate buffer) instead of STZ have a Ps'alb of 3.144 ± 0.295 (cm/s × 10^{-7}).[6] In cardiac perfused mice, kidney sections were stained with FITC-labeled *Lycopersicon esculentum* lectin (LEL) and R18. Confocal representative images (magnification: 63×) (Gi). Top scale bar: 20 μm; bottom scale bar: 5 μm. ROI, region of interest used to measure the distance between the fluorophore profile peaks (peak-to-peak [P-P], Gii). P-P is an index of glycocalyx depth (one-tailed unpaired *t* test, Giii). Kidney sections from a separate batch of vehicle control DBA2/J male mice were subjected to the same processing and staining and show a P-P (132.0 ± 13.43; $n = 5$) significantly higher than the P-P of the STZ group (showing that STZ-induced diabetes reduces eGlx thickness) and non-significantly different from the P-P of STZ + AAV.VEGFC mice (one-way ANOVA, $p < 0.01$, followed by Bonferroni's multiple comparison test, $p < 0.01$). All data are given as mean ± SEM. *$p < 0.05$, **$p < 0.01$, and ***$p < 0.001$.

AAV.neph-VEGFC ameliorates albuminuria and restores the glomerular eGlx in T1D

VEGFC gene therapy was applied to an experimental model of type 1 DKD (Figure 2A). Following streptozotocin (STZ) injection, all mice became hyperglycemic (Figure 2B). At 4 weeks post-STZ, mice received the AAV.neph-*VEGFC* via tail vein injection at 7.50 × 10^{13} genomic copies (GC)/kg, a dose based on Ding et al.[8] The AAV intervention did not affect body weight (Figure S2). *VEGFC* mRNA expression significantly increased in sieved glomeruli, but not in the whole kidney (Figure 2C). Mirroring this, *in situ* hybridization of an AAV regulatory element, also driven by the nephrin promoter, demonstrated transgene expression specifically in the glomerulus (Figures 2Di and 2Dii). Transgene mRNA expression increased significantly in the liver (almost 2,000 times higher in AAV-treated mice) and to a lesser extent in the lung (Figure S3). Resident/infiltrating macrophages were shown to be unchanged between STZ and STZ+AAV.*VEGFC* mice in both glomerular and tubular regions (Figure S4). Of note, very few macrophages were quantified within glomeruli. *VEGFC* gene therapy was expected to prevent albuminuria and glomerular leakiness, as previously shown *ex vivo* and in transgenic mice.[7] Importantly, AAV.neph-*VEGFC* significantly attenuated the diabetes-induced albuminuria

(Figure 2E, fold change due to variability in urine albumin-to-creatinine ratio [uACR]; Figure S5, uACR) and glomerular albumin permeability (Figure 2F), a more sensitive and direct measure of glomerular filtration barrier integrity than uACR.[9]

We hypothesized that *VEGFC* gene therapy would prevent diabetes-induced GEnC barrier dysfunction, specifically through restoration of eGlx damage. EGlx components were labeled with FITC-labeled tomato lectin, and the endothelial membrane was labeled with octadecyl rhodamine B chloride (R18) (Figure 2Gi). A linear region of interest, from the luminal to the abluminal side of the glomerular capillary, provided fluorophore profiles, and the distance between the peaks of these profiles (lectin to R18, respectively) was used as a measure of eGlx depth (Figure 2Gii).[10] EGlx depth was significantly increased in AAV-treated diabetic mice compared to untreated diabetic mice (Figure 2Giii).

DISCUSSION

We demonstrate that gene therapy can be used to target the glomerular filtration barrier in DKD, confirming the effectiveness of *VEGFC* gene therapy in T1D.

DKD is a progressive condition, and there are no available treatments that reverse existing kidney damage. Current pharmacological treatments, such as renin-angiotensin system inhibitors and sodium-glucose co-transporter 2 inhibitors, aim to manage underlying conditions to slow the renal function decline in T2D but have not been approved for patients with T1D. Gene therapies offer promising strategies to address this gap. Herein, we propose a podocyte-directed gene therapy using an AAV2/9 capsid in combination with a podocyte-specific promoter, a gene delivery approach effective in rescuing from podocin defects in models of nephrotic syndrome.[8]

It is well established that paracrine growth factors play crucial roles in the crosstalk between podocytes and the adjacent GEnCs to ensure adequate blood filtration at the glomerular filtration barrier. The VEGFC precursor undergoes a series of proteolytic processing steps that increase its affinity and ability to activate VEGFR3, and the fully processed mature form can also activate VEGFR2, as reported in endothelial cells *in vitro*.[11] VEGFR2, which is expressed on the glomerular endothelium, is activated (phosphorylated) by recombinant VEGFC in cultured GEnCs.[6] Here, we demonstrate that AAV-induced VEGFC is secreted from podocytes and activates VEGFR2 in cultured GEnC, suggesting functional hVEGFC. Previously, we showed that VEGFC increases monolayer integrity in cultured GEnC and counteracts the increased permeability of *ex vivo* glomeruli from T2D *db/db* mice.[5,7] Inducing podocyte-specific VEFGC expression in transgenic mice reduced uACR in STZ-driven diabetes, both prophylactically and as an intervention.[7] Importantly, we have now translated this into a *VEGFC* gene therapy that reduced uACR by ~64%, as well as glomerular permeability, in T1D mice. Of note, a ≥30% reduction in albuminuria is recommended to slow chronic kidney disease progression by the American Diabetes Association.[12]

Podocyte-directed AAV-*VEGFC* also increased the depth of glycocalyx, which is damaged in humans with T1D, including in kidney biopsies, and in STZ-treated mice.[10,13,14] This finding supports the growing evidence that targeting eGlx shedding is an effective strategy to mitigate the increased glomerular permeability and albuminuria in experimental DKD.[9,10] VEGFC enhances the synthesis of sulfated glycosaminoglycans in cultured GEnC and restores impaired glomerular permeability and the eGlx damage caused by glycosaminoglycan-shedding enzymes.[6,7] We have shown that AAV-induced VEGFC increased eGlx depth, thereby improving glomerular endothelial macromolecular barrier properties, leading to an overall reduction in albuminuria and glomerular permeability.

We suggest that chronic podocyte-specific VEGFC overexpression alters glomerular signaling of $VEGFA_{165}$. $VEGFA_{165}$, essential for maintaining the glomerular endothelium and normal blood filtration, is produced by podocytes and acts on GEnC mainly by activating VEGFR2.[15] There are limitations in extrapolating these mouse models to human disease, as rodent models cannot fully recapitulate disease progression or severity. In particular, STZ-induced mice do not develop a reduction in the estimated glomerular filtration rate (eGFR) and therefore loss in renal function. However, increased levels of VEGFA and VEGFR2 are linked to human kidney disease, including early DKD.[16,17] Using two human DKD datasets from the Gene Expression Omnibus database, upregulated VEGFA to VEGFR2 glomerular signaling was shown. Also, VEGFA expression correlated with increased eGFR (hyperfiltration, indicating early disease), but VEGFC did not.[18] Importantly, these human datasets reflect changes that have been shown previously in diabetic rodent models. $VEGFA_{165}$ and mature VEGFC compete for VEGFR2 binding in cultured endothelial cells.[11] In cultured GEnC, recombinant VEGFA phosphorylates VEGFR2 more rapidly and to a higher degree than VEGFC and, unlike VEGFC, increases permeability.[5] VEGFA also increased mouse glomeruli permeability *ex vivo*, which was ameliorated by VEGFC.[7] In addition, and in contrast to VEGFC, VEGFA promoted shedding of charged glycosaminoglycans in cultured GEnC.[6] At week 8, STZ diabetic mice showed increased glomerular VEGFA and VEGFR2 expression (VEGFC was unchanged), but this was attenuated in diabetic mice overexpressing VEGFC in podocytes.[7] In addition to attenuating the diabetes-induced changes in VEGFA signaling, podocyte-specific VEGFC overexpression promoted VEGFR2/VEGFR3 heterodimerization in healthy transgenic mice.[7] Thus, the AAV-induced VEGFC may attenuate $VEGFA_{165}$ signaling by competing for VEGFR2 binding and activating different downstream signals through VEGFR3, resulting in reduced permeability and improved eGlx.

The use of VEGFC clinically has shown potential in breast cancer-related lymphedema using the adenovirus expressing *VEGFC*, Lymphactin. Importantly, we have shown that AAV.neph-*VEGFC* injection into wild-type mice is tolerated, and mice appear macroscopically normal. There are conflicts in the literature regarding VEGFC in disease. Circulating VEGFC has been significantly correlated with weight gain/metabolic syndrome,[19] and overexpression of

VEGFC in mouse skin led to elevated blood glucose and insulin resistance.[20] However, previous work has also shown that circulating VEGFC is significantly inversely related to all-cause mortality, with low levels of serum VEGFC being an independent risk factor for cardiovascular death in patients with coronary artery disease, in a multicenter prospective cohort study (n = 2,418).[21] In our STZ mice, we demonstrated that AAV.neph-*VEGFC* did not affect weight gain or induce hyperglycemia. VEGFC can act as a chemoattractant to macrophages under inflammatory conditions,[22] but targeted delivery of VEGFC reduces immune cell density in diabetic wounds and helps to accelerate repair.[23] We found no evidence of increased macrophage presence in the STZ mice treated with AAV.neph-*VEGFC*, suggesting that glomerular VEGFC does not attract macrophages.

While VEGFA inhibition is of interest in DKD as a potential therapy, it can have undesirable effects.[24] Therapies that attenuate excessive $VEGFA_{165}$ signaling without abolishing its essential functions are attractive. Systemic treatment with recombinant $VEGFA_{165}$b, which binds to VEGFR2 with equal affinity to $VEGFA_{165}$ but activates different downstream signaling in endothelial cells, restored eGlx and ameliorated DKD in mice.[14] Blocking excessive VEGFR2 activation by systemic treatment with either a fragmented VEGFR2 antibody or a VEGFR2 kinase inhibitor has also shown promise in experimental DKD.[25] In contrast to these strategies, our gene therapy approach aims to promote VEGFC signaling specifically in the glomerulus. While the half-life of recombinant VEGFC is short (<15 min) in mouse circulation,[26] our gene therapy approach would overcome this, as AAVs achieve long-term transgene expression. This is primarily attributed to the stabilization of the AAV genome as circular double-stranded extrachromosomal DNA (episomes) that persist inside the nucleus of postmitotic cells, like podocytes. AAVs are also the preferred gene therapy vector for their minimal pathogenicity.

We believe *VEGFC* gene therapy may be particularly beneficial in patients with T1D at the early stage of DKD, when albuminuria is increasing. Although this study focused on DKD, cell-specific *VEGFC* gene therapy could be beneficial in other kidney diseases, such as polycystic kidney disease.[27] We acknowledge that the transgene expression was not exclusive to the glomeruli, as anticipated with the dose and administration route.[8] If a systemic route were to be pursued, then extensive safety profiling would be necessary in terms of liver function and systemic VEGFC expression. However, to avoid potential side effects of off-target expression, the next steps will involve using a direct delivery route with a lower virus titer. Targeted delivery in Gottingen minipigs, using AAV with a podocyte-specific promoter, resulted in glomerular-specific expression, with no extra-renal expression,[28] giving us confidence that off-target expression can be mitigated.

In summary, this study suggests that *VEGFC* gene therapy mitigates the deleterious effects of diabetes on the glomerular filtration barrier and represents a novel and highly promising treatment strategy for DKD in T1D.

MATERIALS AND METHODS

Please refer to supplemental materials and methods.

DATA AND CODE AVAILABILITY

All summary data are included within the manuscript. There are no large datasets. Raw data can be provided upon request.

ACKNOWLEDGMENTS

A.M.A. was funded by BHF PG/20/10187, Kidney Research UK RP_005_20221128, and Elizabeth Blackwell Institute MRC Confidence in Concepts Award 2019/20. C.M. was funded by Diabetes UK (grant 19/0006037). H.S.-C. was funded by a Wellcome Trust Partnership Award Fellowship 2023. M.G. was funded by MRC project grant MR/T031921/1 and Diabetes UK (grant 19/0006037). K.O. was funded by Diabetes UK (grant 19/0006037). R.R. was funded by the British Heart Foundation (grant PG/22/11121). W.Y.D. was funded by an NIHR Clinical Lectureship (grant CL-2019-25-003). The authors gratefully acknowledge the Wolfson Bioimaging Facility and the Animal Services Unit for their support and assistance in this work, and Sevil Erarslan Catak for kindly donating tissue sections. This has previously been submitted in abstract form at the European Society for Microcirculation Conference (2023), UK Kidney Week (2023), and the Annual Conference of the British Microcirculation and Vascular Biology Society (2023).

AUTHOR CONTRIBUTIONS

Data curation, A.M.A., C.M., H.S.-C., H.W., M.G., K.O., and R.R.; formal analysis, A.M.A., C.M., H.S.-C., H.W., M.G., K.O., and R.R.; investigation, A.M.A. and C.M.; writing – original draft, A.M.A.; writing – review & editing, C.M., H.S.-C., H.W., M.G., K.O., R.R., W.Y.D., G.I.W., S.C.S., and R.R.F.; methodology, W.Y.D.; resources, W.Y.D.; supervision, G.I.W., S.C.S., and R.R.F.; conceptualization, R.R.F.; funding acquisition, R.R.F.; project administration, R.R.F.

DECLARATION OF INTERESTS

The authors have nothing to declare.

SUPPLEMENTAL INFORMATION

Supplemental information can be found online at https://doi.org/10.1016/j.ymthe.2025.10.001.

REFERENCES

1. Zhou, B., Rayner, A.W., Gregg, E.W., Sheffer, K.E., Carrillo-Larco, R.M., Bennett, J.E., Shaw, J.E., Paciorek, C.J., Singleton, R.K., Pires, A.B., and Stevens, G.A. (2024). Worldwide trends in diabetes prevalence and treatment from 1990 to 2022: a pooled analysis of 1108 population-representative studies with 141 million participants. Lancet *404*, 2077–2093. https://doi.org/10.1016/s0140-6736(24)02317-1.
2. Caramori, M.L., Kim, Y., Huang, C., Fish, A.J., Rich, S.S., Miller, M.E., Russell, G., and Mauer, M. (2002). Cellular basis of diabetic nephropathy: 1. Study design and renal structural-functional relationships in patients with long-standing type 1 diabetes. Diabetes *51*, 506–513. https://doi.org/10.2337/diabetes.51.2.506.
3. Morrish, N.J., Wang, S.L., Stevens, L.K., Fuller, J.H., and Keen, H. (2001). Mortality and causes of death in the WHO Multinational Study of Vascular Disease in Diabetes. Diabetologia *44*, S14–S21. https://doi.org/10.1007/pl00002934.
4. Satchell, S.C., and Tooke, J.E. (2008). What is the mechanism of microalbuminuria in diabetes: a role for the glomerular endothelium? Diabetologia *51*, 714–725.
5. Foster, R.R., Slater, S.C., Seckley, J., Kerjaschki, D., Bates, D.O., Mathieson, P.W., and Satchell, S.C. (2008). Vascular endothelial growth factor-C, a potential paracrine regulator of glomerular permeability, increases glomerular endothelial cell monolayer integrity and intracellular calcium. Am. J. Pathol. *173*, 938–948.
6. Foster, R.R., Armstrong, L., Baker, S., Wong, D.W.L., Wylie, E.C., Ramnath, R., Jenkins, R., Singh, A., Steadman, R., Welsh, G.I., et al. (2013). Glycosaminoglycan regulation by VEGFA and VEGFC of the glomerular microvascular endothelial cell glycocalyx in vitro. Am. J. Pathol. *183*, 604–616. https://doi.org/10.1016/j.ajpath.2013.04.019.

7. Onions, K.L., Gamez, M., Buckner, N.R., Baker, S.L., Betteridge, K.B., Desideri, S., Dallyn, B.P., Ramnath, R.D., Neal, C.R., Farmer, L.K., et al. (2019). VEGFC Reduces Glomerular Albumin Permeability and Protects Against Alterations in VEGF Receptor Expression in Diabetic Nephropathy. Diabetes *68*, 172–187. https://doi.org/10.2337/db18-0045.

8. Ding, W.Y., Kuzmuk, V., Hunter, S., Lay, A., Hayes, B., Beesley, M., Rollason, R., Hurcombe, J.A., Barrington, F., Masson, C., et al. (2023). Adeno-associated virus gene therapy prevents progression of kidney disease in genetic models of nephrotic syndrome. Sci. Transl. Med. *15*, eabc8226. https://doi.org/10.1126/scitranslmed.abc8226.

9. Desideri, S., Onions, K.L., Qiu, Y., Ramnath, R.D., Butler, M.J., Neal, C.R., King, M. L.R., Salmon, A.E., Saleem, M.A., Welsh, G.I., et al. (2018). A novel assay provides sensitive measurement of physiologically relevant changes in albumin permeability in isolated human and rodent glomeruli. Kidney Int. *93*, 1086–1097. https://doi.org/10.1016/j.kint.2017.12.003.

10. Crompton, M., Ferguson, J.K., Ramnath, R.D., Onions, K.L., Ogier, A.S., Gamez, M., Down, C.J., Skinner, L., Wong, K.H., Dixon, L.K., et al. (2023). Mineralocorticoid receptor antagonism in diabetes reduces albuminuria by preserving the glomerular endothelial glycocalyx. JCI Insight *8*, e154164. https://doi.org/10.1172/jci.insight.154164.

11. Joukov, V., Sorsa, T., Kumar, V., Jeltsch, M., Claesson-Welsh, L., Cao, Y., Saksela, O., Kalkkinen, N., and Alitalo, K. (1997). Proteolytic processing regulates receptor specificity and activity of VEGF-C. EMBO J. *16*, 3898–3911.

12. American Diabetes Association Professional Practice Committee (2025). Chronic Kidney Disease and Risk Management: Standards of Care in Diabetes-2025. Diabetes Care *48*, S239–S251. https://doi.org/10.2337/dc25-S011.

13. Nieuwdorp, M., Mooij, H.L., Kroon, J., Atasever, B., Spaan, J.A.E., Ince, C., Holleman, F., Diamant, M., Heine, R.J., Hoekstra, J.B.L., et al. (2006). Endothelial glycocalyx damage coincides with microalbuminuria in type 1 diabetes. Diabetes *55*, 1127–1132.

14. Oltean, S., Qiu, Y., Ferguson, J.K., Stevens, M., Neal, C., Russell, A., Kaura, A., Arkill, K.P., Harris, K., Symonds, C., et al. (2015). Vascular Endothelial Growth Factor-A165b Is Protective and Restores Endothelial Glycocalyx in Diabetic Nephropathy. J. Am. Soc. Nephrol. *26*, 1889–1904. https://doi.org/10.1681/asn.2014040350.

15. Sison, K., Eremina, V., Baelde, H., Min, W., Hirashima, M., Fantus, I.G., and Quaggin, S.E. (2010). Glomerular structure and function require paracrine, not autocrine, VEGF-VEGFR-2 signaling. J. Am. Soc. Nephrol. *21*, 1691–1701. https://doi.org/10.1681/asn.2010030295.

16. Cooper, M.E., Vranes, D., Youssef, S., Stacker, S.A., Cox, A.J., Rizkalla, B., Casley, D. J., Bach, L.A., Kelly, D.J., and Gilbert, R.E. (1999). Increased renal expression of vascular endothelial growth factor (VEGF) and its receptor VEGFR-2 in experimental diabetes. Diabetes *48*, 2229–2239.

17. Nussdorfer, P., Petrovič, D., Alibegović, A., Cilenšek, I., and Petrovič, D. (2024). The KDR Gene rs2071559 and the VEGF Gene rs6921438 May Be Associated with Diabetic Nephropathy in Caucasians with Type 2 Diabetes Mellitus. Int. J. Mol. Sci. *25*, 9439. https://doi.org/10.3390/ijms25179439.

18. Zhang, L., Wang, Z., Tang, F., Wu, M., Pan, Y., Bai, S., Lu, B., Zhong, S., and Xie, Y. (2024). Identification of Senescence-Associated Biomarkers in Diabetic Glomerulopathy Using Integrated Bioinformatics Analysis. J. Diabetes Res. *2024*, 5560922. https://doi.org/10.1155/2024/5560922.

19. Zafar, M.I., Mills, K., Ye, X., Blakely, B., Min, J., Kong, W., Zhang, N., Gou, L., Regmi, A., Hu, S.Q., et al. (2018). Association between the expression of vascular endothelial growth factors and metabolic syndrome or its components: a systematic review and meta-analysis. Diabetol. Metab. Syndr. *10*, 62. https://doi.org/10.1186/s13098-018-0363-0.

20. Karaman, S., Hollmén, M., Yoon, S.Y., Alkan, H.F., Alitalo, K., Wolfrum, C., and Detmar, M. (2016). Transgenic overexpression of VEGF-C induces weight gain and insulin resistance in mice. Sci. Rep. *6*, 31566. https://doi.org/10.1038/srep31566.

21. Wada, H., Suzuki, M., Matsuda, M., Ajiro, Y., Shinozaki, T., Sakagami, S., Yonezawa, K., Shimizu, M., Funada, J., Takenaka, T., et al. (2018). VEGF-C and Mortality in Patients With Suspected or Known Coronary Artery Disease. J. Am. Heart Assoc. *7*, e010355. https://doi.org/10.1161/jaha.118.010355.

22. Karaman, S., Hollmén, M., Robciuc, M.R., Alitalo, A., Nurmi, H., Morf, B., Buschle, D., Alkan, H.F., Ochsenbein, A.M., Alitalo, K., et al. (2015). Blockade of VEGF-C and VEGF-D modulates adipose tissue inflammation and improves metabolic parameters under high-fat diet. Mol. Metab. *4*, 93–105. https://doi.org/10.1016/j.molmet.2014.11.006.

23. Brunner, L.M., He, Y., Cousin, N., Scholl, J., Albin, L.K., Schmucki, B., Supersaxo, S., Restivo, G., Hafner, J., Neri, D., et al. (2023). Promotion of Lymphangiogenesis by Targeted Delivery of VEGF-C Improves Diabetic Wound Healing. Cells *12*, 472. https://doi.org/10.3390/cells12030472.

24. Eremina, V., Jefferson, J.A., Kowalewska, J., Hochster, H., Haas, M., Weisstuch, J., Richardson, C., Kopp, J.B., Kabir, M.G., Backx, P.H., et al. (2008). VEGF inhibition and renal thrombotic microangiopathy. N. Engl. J. Med. *358*, 1129–1136.

25. Liu, D., Song, Y., Chen, H., You, Y., Zhu, L., Zhang, J., Xu, X., Hu, J., Huang, X., Wu, X., et al. (2023). Anti-VEGFR2 F(ab')(2) drug conjugate promotes renal accumulation and glomerular repair in diabetic nephropathy. Nat. Commun. *14*, 8268. https://doi.org/10.1038/s41467-023-43847-2.

26. Veikkola, T., Jussila, L., Makinen, T., Karpanen, T., Jeltsch, M., Petrova, T.V., Kubo, H., Thurston, G., McDonald, D.M., Achen, M.G., et al. (2001). Signalling via vascular endothelial growth factor receptor-3 is sufficient for lymphangiogenesis in transgenic mice. EMBO J. *20*, 1223–1231. https://doi.org/10.1093/emboj/20.6.1223.

27. Huang, J.L., Woolf, A.S., Kolatsi-Joannou, M., Baluk, P., Sandford, R.N., Peters, D.J. M., McDonald, D.M., Price, K.L., Winyard, P.J.D., and Long, D.A. (2016). Vascular Endothelial Growth Factor C for Polycystic Kidney Diseases. J. Am. Soc. Nephrol. *27*, 69–77. https://doi.org/10.1681/asn.2014090856.

28. Illingworth, S., Gurung, K., Kuzmuk, V., Wood, H., Asfahani, R., Gennari, A., Ding, W., Taliotis, D., Welsh, G., Brown, A., and Saleem, M. (2024). #1118 Direct delivery of novel gene therapy to podocytes enables pathway to clinical translation for the treatment of glomerular diseases. Nephrol. Dial. Transpl. *39*, gfae069-0032. https://doi.org/10.1093/ndt/gfae069.032.

YMTHE, Volume *34*

Supplemental Information

Podocyte-directed *VEGFC* gene therapy prevents increased glomerular permeability and glycocalyx damage in experimental type 1 diabetes

Aldara Martin Alonso, Carl J. May, Holly Stowell-Connolly, Haijie Wu, Monica Gamez, Khadija Ourradi, Raina D. Ramnath, Wen Yi Ding, Gavin I. Welsh, Simon C. Satchell, and Rebecca R. Foster

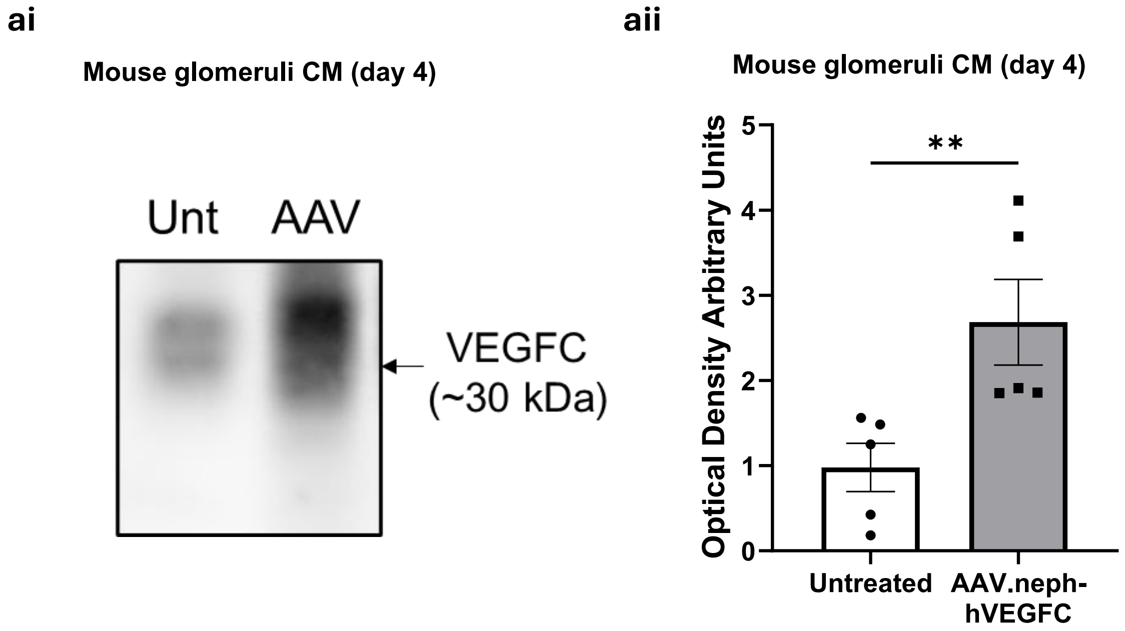

Figure S1 | VEGFC expression in conditioned media of mouse glomeruli at day 4 after *ex vivo* transduction with AAV.neph-*VEGFC*. Isolated murine glomeruli from wild type SV129 mice were transduced with AAV.neph-*VEGFC* (MOI 1 x 10^5; manufactured by VectorBuilder) and expression of hVEGFC was measured at day 4 after *ex vivo* infection by western blotting of the conditioned media (CM) (ai). Optical density arbitrary units (aii; Untreated, n = 5; AAV.neph-*VEGFC*, n = 5 (one-tailed unpaired t test)). All data are presented as mean ± SEM. $**p < 0.01$.

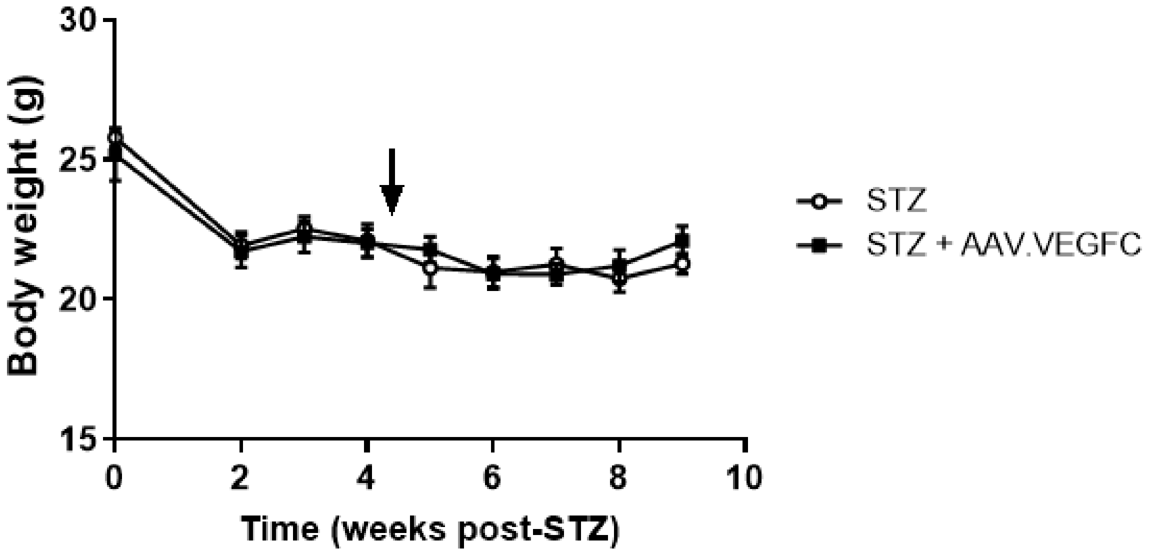

Figure S2 | Body weight of type 1 diabetic mice does not change with AAV.neph-*VEGFC* intervention. Body weight overtime post-STZ administration. The arrow indicates time of AAV.neph-*VEGFC* injection (AAV.*VEGFC*). STZ, n = 8 animals (n = 7 at week 9); STZ + AAV.neph-*VEGFC*, n = 9 animals. All data are presented as mean ± SEM.

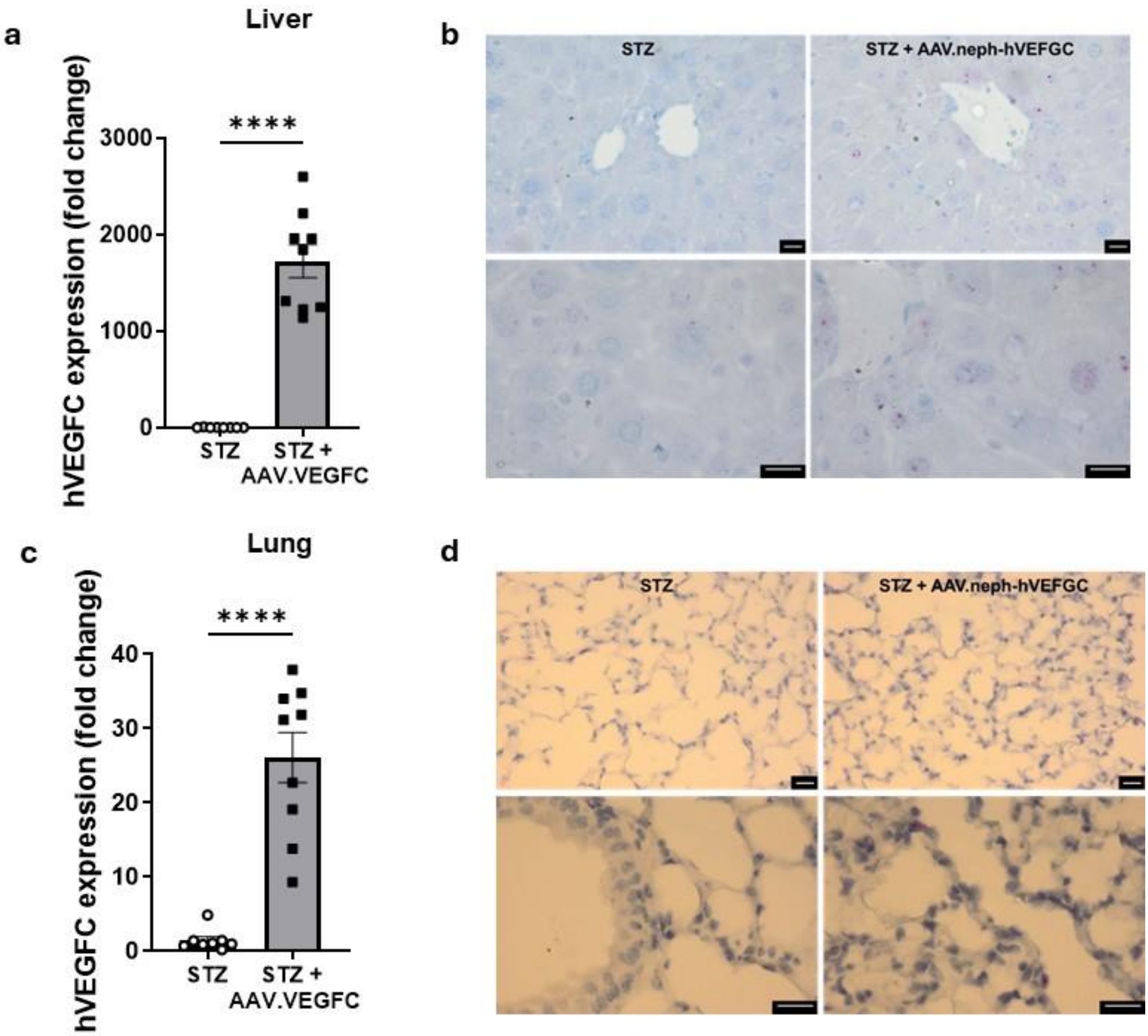

Figure S3 | Transgene expression in other tissues of type 1 diabetic mice treated with AAV.neph-*VEGFC*. a) Human VEGFC mRNA expression in liver tissue. STZ, n = 8 animals; STZ + AAV.neph-*VEGFC*, n = 9 animals. One-tailed unpaired t test. b) Representative images of chromogenic (red) in situ hybridization of WPRE in liver sections. Magnifications 20x (top) and 40x (bottom). Scale bar: 20 µm. c) Human VEGFC mRNA expression in lung tissue. STZ, n = 8 animals; STZ + AAV.neph-*VEGFC*, n = 9 animals. One-tailed unpaired t test. d) Representative images of chromogenic (red) in situ hybridization of WPRE in lung sections. Magnifications 20x (top) and 40x (bottom). Scale bar: 20 µm. ****$P < 0.0001$. All data are presented as mean ± SEM.

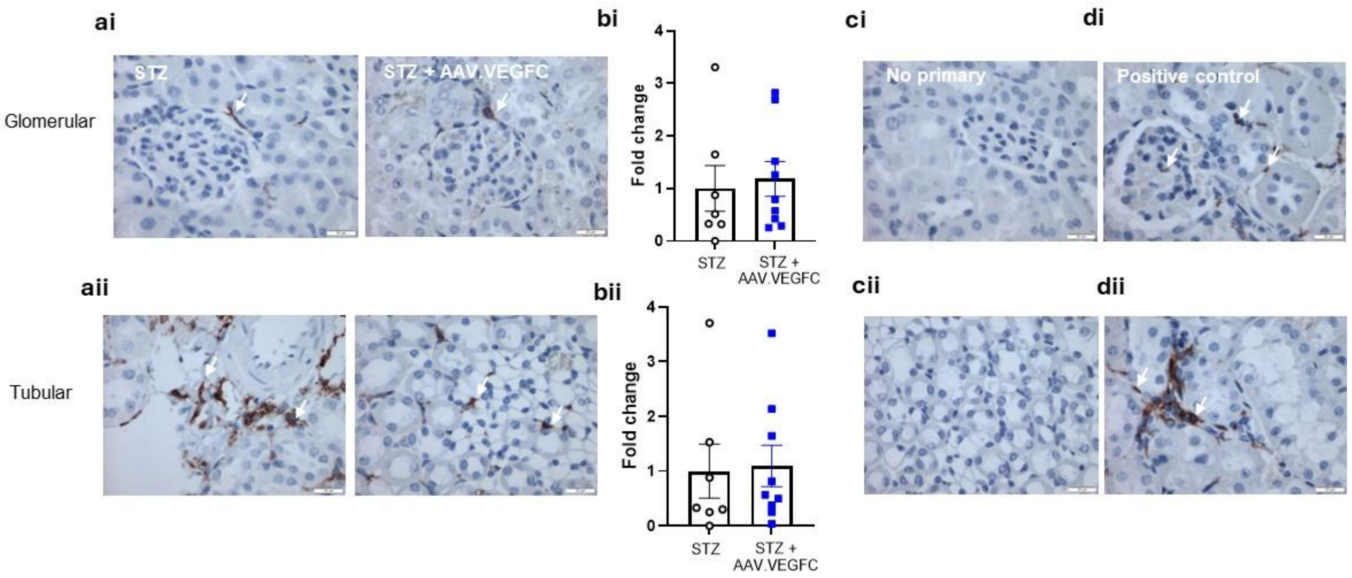

Figure S4 | AAV.neph-*VEGFC* does not impact on glomerular or tubular macrophage staining. (a) Representative images of F4/80 immunohistochemistry staining in glomerular (ai) and tubular (aii) areas of the kidney from STZ (n = 8) and STZ + AAV.neph-*VEGFC* (AAV.*VEGFC*) (n = 9) mice. (b) Quantification of F4/80 staining using integrated density and normalised to the STZ group for glomerular (bi) and tubular (bii) staining. (c) Representative images showing no primary antibody negative control in glomerular (ci) and tubular (cii) regions. (d) Representative images of tissue sections from a Type 2 mouse model of diabetes at 16 wk for glomerular (di) and tubular (dii) staining. White arrows indicate positive staining. Scale bar: 20 µm. All data are presented as mean ± SEM.

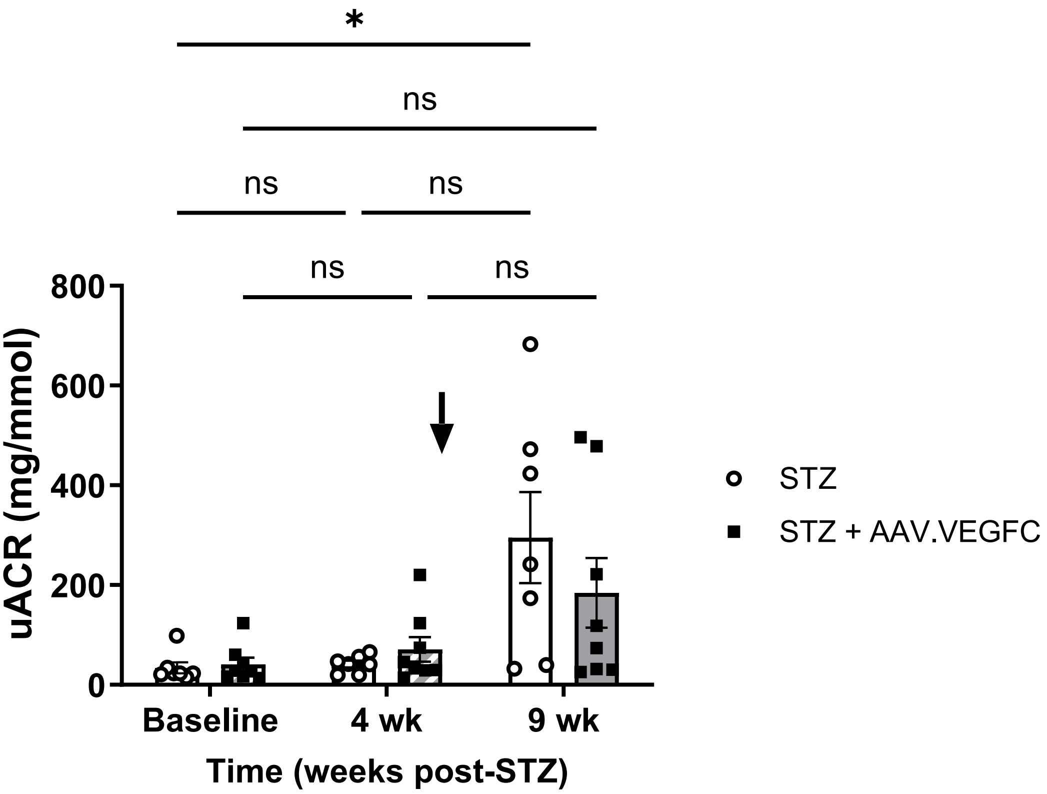

Figure S5 | Urine albumin-to-creatinine ratio of type 1 diabetic mice treated with AAV.neph-*hVEGFC*. Absolute uACR after injection of AAV.neph-*VEGFC* (arrow). Two-way ANOVA followed by Dunnett's multiple comparison test. STZ, n = 7 animals; STZ + AAV.neph-*hVEGFC*, n = 8 animals. *P < 0.05. All data are presented as mean ± SEM.

Supplementary Materials and Methods

Virus production

Initially, the AAV particles used were produced in house. The Flag-tagged human *VEGFC* gene (NCBI Ref Seq: NM_005429.2, Sino Biologicals) was cloned into an AAV9 plasmid, which contains the minimal human nephrin promoter (provided by Dr Wen Ding) and AAV2 ITRs, using *AflII* and *SbfI* restriction sites. Viral particles were produced in HEK293T by triple transfection with the AAV plasmid (the helper plasmid with adenoviral genes (pHGTI-Adeno1) and the capsid plasmid as previously described.[1] The capsid AAV9 sequence was used to target mouse podocytes.[1] The AAV preparation was purified by iodixanol gradient ultra-centrifugation using a Type70.1 Ti rotor, concentrated in phosphate-buffered saline (PBS) and titrated by alkaline gel electrophoresis and qPCR targeting the ITR sequence. Later, AAV manufacturing was outsourced (VectorBuilder).

Cell culture

Podocyte and glomerular endothelial cell lines used in this work are conditionally immortalised cell lines generated in house and cultured as previously described.[2-4] These cell lines were immortalised with a SV40 temperature-sensitive T antigen. Cell lines proliferate at 33°C and, when switched to 37°C, they become differentiated. Both human and mouse podocytes were cultured with RPMI-1640 containing L-glutamine and $NaHCO_3$ supplemented with 10% FBS. Human conditionally immortalised glomerular endothelial cells (GEnC) were cultured with the EBM-2 Endothelial Cell Growth Basal Medium-2 supplemented with EGM-2 Endothelial Cell Growth Medium-2 BulletKit (Lonza), except for the GA-1000. Conditionally immortalised podocytes at a confluence of 30-50% were transduced with AAV and thermoswitched to 37°C. For VEGFC expression, podocytes were used 10-20 days after transduction, when cells are fully differentiated.

RNA extraction and quantitative PCR

RNA was extracted using Tri reagent (Sigma Aldrich) and treated with RQ1 DNAse kit (Promega) following supplier instructions. cDNA was synthesized using a High-Capacity RNA-

to-cDNA kit (Thermo Fisher Scientific). Quantitative qPCR was performed in triplicates using TaqMan Fast Advanced Master Mix (Thermo Fisher Scientific) and TaqMan Gene Expression Assays (Thermo Fisher Scientific; Human VEGFC: Hs00153458_m1; Mouse GAPDH: Mm99999915_g1; Human GAPDH: Hs02786624_g1) following supplier instructions in a StepOnePlus Real-Time PCR System (Applied Biosystems). The fold change was calculated using the $2^{-\Delta\Delta Ct}$ method.

Western blotting

Protein lysates were subjected to SDS-PAGE under reducing conditions and transferred to a PVDF membrane. Membranes were blocked and incubated with primary antibodies overnight at 4°C (anti-human VEGFC (Cat. MA5-26494, Thermo Fisher Scientific) at 1:200; anti-pan phosphotyrosine antibody (cat. SAB5700563, Sigma-Aldrich) at 1:1000; anti-VEGFR2 (cat. AF357, R&D) at 1:1000) before washing and incubation with HRP-conjugated secondary antibodies. After adding the Clarity ECL Western Blotting substrate (Bio-Rad), bands were visualised in an Amersham Imager 600 (GE Healthcare Life Sciences) and quantified in ImageJ (NIH).

Immunoprecipitation

Immunoprecipitation was performed using the Thermo Scientific Pierce™ Classic IP kit (cat. 26146) and the anti-VEGFR2 (cat. 55B11, Cell Signalling Technologies). Then samples were subjected to western blotting as described above.

Primary glomeruli culture

Glomeruli were sieved from kidney cortex as previously described.[3] Human glomeruli were isolated from kidneys that were unsuitable for transplantation. Use of human kidney tissue was approved by national and local research ethics committees and conducted in accordance with the tenets of the Declaration of Helsinki. For mouse glomeruli, a yield of ~8000 glomeruli was obtained from two adult mouse kidneys. Glomeruli were cultured in supplemented EBM-2 medium.

Animals

All animal experiments and procedures were approved by the UK Home Office and approved locally by AWERB committee. In this study, *n* refers to the number of animals. Animals were maintained in a controlled environment (21-24°C and 12:12 h light-dark cycle). Animals were acclimatised for 1 week after arrival. Standard laboratory chow and drinking water were provided *ad libitum* (unless otherwise required for experimental reasons). We carried out an initial pilot study of 6 wk old male wild type C57BL/6 mice, given 1.5×10^{12} AAV.*VEGFC* by tail vein injection or left untreated (n=3 each group). Urine was collected weekly for 5 wk. Glomerular hVEGFC mRNA was confirmed in n=2 animals (13.5 ± 7.4 fold increase). Mice were macroscopically normal and uACR remained unchanged (untreated: 11.1 ± 5.8 AAV.*VEGFC*: 11.9 ± 3.2 mg/mmol), confirming that AAV.*VEGFC* could be tolerated well and was not harmful. In this study, we used a model of early diabetic kidney disease.[5] Diabetes was induced in a total of 18 10-week-old DBA2/J male mice (Charles River) by intraperitoneal injection of streptozotocin (STZ) following the low-dose STZ protocol (50 mg/kg over 5 consecutive days) from the *Diabetic Complications Consortium*. Blood glucose was measured by tail-tip blood droplet analysis using a glucometer (GlucoRx Nexus). Body weight was regularly monitored. STZ-injected mice were considered diabetic when blood glucose levels were ≥ 15 mmol/l. Urine was collected by placing mice in cages containing LabSand (Braintree Scientific, Inc.) for up to 3 hours. Four weeks after STZ administration, a subgroup (n=9) mice received AAV-*VEGFC* particles (custom made by VectorBuilder) via tail vein injection at a dose of 7.50×10^{13} GC/kg. The random allocation of mice to the AAV treatment group was done using the RAND function in Microsoft Excel while ensuring groups were balanced per cage and that body weight and blood glucose were balance between groups. The sample size accounted for the success rate and mortality rates of the method used to induce albuminuria (primary outcome) and on success rate of tail vein injection. Not all animals were used for all outcomes because of the diabetes adverse effects. Humane endpoints were animals showing overt signs of ill-health and/ or weight loss of >20% from baseline, and which did not improve in response to supportive measures (hydration, heat pad). Two animals were terminated by schedule 1 due to diabetic complications at endpoint. Mice were terminated 9 or 10 weeks after STZ

administration. Tissue was collected and either snap frozen in liquid nitrogen, or fixed. Some mice were cardiac perfused with Ringer solution (NaCl, 132mM; KCl, 4.6mM; $MgSO_4$-$7H_2O$ 1.27mM; $CaCl_2$-$2H_2O$ 2mM; $NaHCO_3$, 25mM; D(+)glucose, 5.5mM; N-2-hydroxyethylpiperazine-N'-2-ethanesulphonic (HEPES) acid, 3.07mM; HEPES sodium salt, 1.9mM, pH 7.40) before termination and then tissue collected. Glomeruli were isolated from perfused kidney pieces by graded sieving and collected from the 75 µm pore sieve. Then, glomeruli were either snap frozen or used in the glomerular albumin permeability assay. The investigator was aware of the treatment groups during the experiment but blinded for data analysis.

Urinary albumin-to-creatinine ratio (uACR)

Urinary albumin was quantified using a mouse albumin ELISA (Bethyl Laboratories Inc.) following manufacturer's instructions. Creatinine was measured by the Bristol Langford Clinical Veterinary Service. If uACR was abnormally high, animals were excluded from urinary albumin-creatinine ratio and glomerular albumin permeability (Ps'alb) assay data analysis.

Glomerular albumin permeability assay

The glomerular albumin permeability (Ps'alb) assay was performed as previously described.[6] Briefly, after cardiac perfusing animals with Ringer buffer, kidneys were isolated by graded sieving with 4% BSA in Ringer solution. Glomeruli were incubated with 36.5 µg/ml of R18 (Thermo Fisher Scientific) for 15 min on ice, washed twice in BSA/Ringer and incubated with 30 µg/mL Alexa Fluor 488-BSA (Thermo Fisher Scientific) for 15 min on ice. An individual glomerulus was trapped on a custom-made dish under a Nikon Ti-E inverted confocal microscope (Nikon Instruments, Inc.). While imaging, the perfusate was switched from Alexa Fluor 488-BSA to unlabelled BSA. The rate of decrease in fluorescence intensity within the loop of the capillaries was quantified blinded using the NIS Elements Analysis software (Nikon Instruments, Inc.) and used to calculate Ps'alb.

***In situ* hybridization**

Tissue was fixed in 10% neutral buffered formalin for 16-32 hours at room temperature, paraffin-embedded and sectioned. Sections were probed for Woodchuck Hepatitis Virus

Posttranscriptional Regulatory Element (WPRE) using the RNAscope® 2.5 High-Definition Red Assay (ACD-Biotechne). The manufacturer's instructions were followed with some changes. All sections were subjected to a 30-minute incubation in 0.3M HCl following the deparaffinisation steps. Target retrieval duration was 15 min in kidney and lung sections, while 30 min in liver sections. All sections were incubated in Protease Plus for 30 minutes at 40°C. After incubation with RNAscope® probe WPRE-O2 (2 hrs at 40°C), sections were kept in SSC buffer overnight and the assay continued the following day. Incubation in AMP5 was 90 min at room temperature.

Imaging was performed in Leica DMI6000 widefield microscope with motorised stage and DFC420C camera using the Leica LASX acquisition software or in a Leica DMLB microscope with an Olympus Colorview I camera and Olympus CellSens Entry v1.15 software.

For quantification in kidney sections, images of glomeruli were randomly obtained by acquiring tile scans and the ImageJ (NIH) PlugIn 'Colour Deconvolution' was used. A threshold was set to define positive-WPRE and background staining, respectively, and images were converted to a binary mask. Staining area in the glomerulus was measured by defining a region of interest using the original images, and the mean WPRE staining was normalised to the mean background stain.

Lectin staining

Paraffin-embedded kidney sections (3 µm) were dewaxed in xylene for 10 mins followed by rehydration in graded ethanol and washing in distilled water. After three washes with PBS-tween 0.1% (pH 7.4), sections were incubated with 1% BSA in PBS-tween 0.1% (pH 7.4) for 1 h and wash again. Sections were incubated with FITC-labelled *Lycopersicon esculentum* lectin (LEL-FITC; Sigma-Aldrich) at 1:100 in 1% BSA in PBS-tween 0.5% (pH 6.8) overnight at 4°C. After washing twice with PBS-tween 0.1%, sections were incubated with 300 nM DAPI (Sigma-Aldrich) for 10 min. After washing twice with PBS-tween 0.1% (pH 7.4), sections were incubated for 15 min with Octadecyl Rhodamine B Chloride (R18; Thermo Fisher Scientific) diluted 1:1000 in PBS. After dipping the slides 5 times in PBS, coverslips were mounted using

Vectashield (Vector Laboratories). Sections were imaged using a Leica SP8 AOBS confocal laser scanning microscope attached to a Leica DM I8 inverted epifluorescence microscope.

Endothelial glycocalyx depth analysis

The distance between the peak signals from the LEL-FITC and R18 fluorophores (peak-to-peak) is an index of glycocalyx depth. Peak-to-peak measurements were performed blinded using an ImageJ (NIH) macro.[7] The mean was determined from 360 lines per capillary loop, 1-4 loops per glomerulus, and 4-7 glomeruli per mouse.

Macrophage staining and quantification

Fresh kidney cortex was fixed in 4% PFA overnight and stored in 70% ethanol. Tissues were embedded in paraffin and 5 µm sections were cut. Sections were dewaxed with xylene and hydrated with ethanol (100%, 90% and 70%) and distilled water. Antigen retrieval was performed using 10 mM sodium citrate tribasic buffer (pH 6.0), followed by inactivation of endogenous peroxidase with hydrogen peroxide (3 wt.%, 88597, Merck, Darmstadt, Germany). After blocking with 5% normal goat serum (ab7481, Abcam, Cambridge, UK) in TBST, the sections were incubated with F4/80 (1:400, 70076T, Cell Signalling Technology, Leiden, Netherlands) overnight at 4 °C. After 3 washes with TBST, sections were incubated with HRP reagent (SignalStain® Boost Detection Reagent, 8114P, Cell Signalling Technology, Leiden, Netherlands) for 30 minutes at room temperature. The positive staining was apparent with SignalStain® DAB Substrate Kit (8059P, Cell Signalling Technology, Leiden, Netherlands). Sections were counterstained with hematoxylin for 10 seconds and dehydrated with ethanol and xylene. DPX Mountant (06522, Sigma-Aldrich, Gillingham, UK) was used to seal the sections with coverslips. Images were taken using a Leica DMLB Microscope (Leica Microsystems, Milton Keynes, UK) with an Olympus Colorview I camera and Olympus cellSens Entry v1.15 software. Macrophage staining was quantified by mean % positive stained area in glomerular and tubular regions via ImageJ.

Statistical analysis

Data are expressed as mean ± SEM. Prism 10 software (GrapPad Software, LLC) was used for analysis. Normality tests were performed. Statistical tests used to evaluate changes are

indicated in figure legends. A *P* value of < 0.05 was considered to indicate a significant difference.

References

1. Ding, WY, Kuzmuk, V, Hunter, S, Lay, A, Hayes, B, Beesley, M, Rollason, R, Hurcombe, JA, Barrington, F, Masson, C, Cathery, W, May, C, Tuffin, J, Roberts, T, Mollet, G, Chu, CJ, McIntosh, J, Coward, RJ, Antignac, C, Nathwani, A, Welsh, GI, Saleem, MA: Adeno-associated virus gene therapy prevents progression of kidney disease in genetic models of nephrotic syndrome. *Sci Transl Med,* 15**:** eabc8226, 2023.
2. Satchell, SC, Tasman, CH, Singh, A, Ni, L, Geelen, J, von Ruhland, CJ, O'Hare, MJ, Saleem, MA, van den Heuvel, LP, Mathieson, PW: Conditionally immortalized human glomerular endothelial cells expressing fenestrations in response to VEGF. *Kidney Int,* 69**:** 1633-1640, 2006.
3. Saleem, MA, O'Hare, MJ, Reiser, J, Coward, RJ, Inward, CD, Farren, T, Xing, CY, Ni, L, Mathieson, PW, Mundel, P: A conditionally immortalized human podocyte cell line demonstrating nephrin and podocin expression. *J Am Soc Nephrol,* 13**:** 630-638, 2002.
4. Keir, LS, Firth, R, May, C, Ni, L, Welsh, GI, Saleem, MA: Generating conditionally immortalised podocyte cell lines from wild-type mice. *Nephron,* 129**:** 128-136, 2015.
5. Onions, KL, Gamez, M, Buckner, NR, Baker, SL, Betteridge, KB, Desideri, S, Dallyn, BP, Ramnath, RD, Neal, CR, Farmer, LK, Mathieson, PW, Gnudi, L, Alitalo, K, Bates, DO, Salmon, AHJ, Welsh, GI, Satchell, SC, Foster, RR: VEGFC Reduces Glomerular Albumin Permeability and Protects Against Alterations in VEGF Receptor Expression in Diabetic Nephropathy. *Diabetes,* 68**:** 172-187, 2019.
6. Desideri, S, Onions, KL, Qiu, Y, Ramnath, RD, Butler, MJ, Neal, CR, King, MLR, Salmon, AE, Saleem, MA, Welsh, GI, Michel, CC, Satchell, SC, Salmon, AHJ, Foster, RR: A novel assay provides sensitive measurement of physiologically relevant changes in albumin permeability in isolated human and rodent glomeruli. *Kidney Int,* 93**:** 1086-1097, 2018.
7. Crompton, M, Ferguson, JK, Ramnath, R, Onions, KL, Ogier, AS, Gamez, M, Down, CJ, Skinner, LJ, Wong, KH, Dixon, LK, Sutak, J, Harper, SJ, Pontrelli, P, Gesualdo, L, Heerspink, HL, Toto, RD, Welsh, GI, Foster, RR, Satchell, SC, Butler, MJ: Mineralocorticoid receptor antagonism in diabetes reduces albuminuria by preserving the glomerular endothelial glycocalyx. *JCI insight*, 2023.
